# Tobacco advertising, promotion, and sponsorship ban adoption: A pilot study of the reporting challenges faced by low- and middle-income countries

**DOI:** 10.18332/tid/155816

**Published:** 2023-01-23

**Authors:** Arsenios Tselengidis, Sarah Dance, Sally Adams, Becky Freeman, Jo Cranwell

**Affiliations:** 1 Department for Health, University of Bath, Bath, United Kingdom; 2 Tobacco Control Research Group, University of Bath, Bath, United Kingdom; 3 Institute for Mental Health, University of Birmingham, Birmingham, United Kingdom; 4 Sydney School of Public Health, Faculty of Medicine and Health, The University of Sydney, Sydney, Australia

**Keywords:** WHO FCTC, Article 13, tobacco control, tobacco advertising, low- and middle-income countries

## Abstract

**INTRODUCTION:**

The WHO Framework Convention for Tobacco Control (FCTC) Secretariat has identified issues with Article 13 (Tobacco Advertising, Promotion and Sponsorship) Party policy progress reporting, whilst some researchers remain skeptical of the completeness and accuracy of the data collected as part of the required reporting questionnaire. Gaining a deeper understanding of the challenges encountered when completing these questionnaires could provide insights to improve WHO FCTC progress reporting.

**METHODS:**

Qualitative semi-structured interviews were conducted between January and June 2021, with nine national tobacco control focal point (NFP) individuals (designates who report on WHO FCTC implementation on the Party’s behalf) from low- and middle-income countries. The study analysis used a thematic framework approach involving data familiarization, thematic framework construction, indexing and refining, mapping and interpretation of the results.

**RESULTS:**

The analysis generated four themes: 1) use of different resources, 2) presence of compounding complexities, 3) use of supporting mechanisms employed for tackling the challenges, and 4) recommendations for refinements within the questionnaire and for those completing it.

**CONCLUSIONS:**

The WHO FCTC reporting questionnaire needs improvements that could be piloted and discussed between the Convention Secretariat and the Parties prior to wide scale implementation.

## INTRODUCTION

The World Health Organization Framework Convention on Tobacco Control (WHO FCTC)^1^ is one of the most universally adopted global treaties, covering more than 90% of the global population^2^. Amongst its provisions, it requires the Parties (WHO member states that have ratified the Convention) to comprehensively ban tobacco advertising, promotion and sponsorship (TAPS) (Article 13) and to biennially report their implementation progress to the WHO FCTC Secretariat (Article 21)^1^ – the administrative body responsible for assisting the Parties to fulfil their obligations under the Convention.

### Article 13

Under Article 13 (tobacco advertising, promotion and sponsorship)^1^, Parties have six main obligations: prohibit deceptive promotion of tobacco products; comprehensively ban TAPS in the media; put health warnings on all TAPS; prohibit tobacco sponsorship of international events and/or the participation therein; restrict direct or indirect incentives encouraging tobacco product purchase; and, in case of a non-comprehensive ban adoption, require the disclosure to the governmental authorities of the tobacco industry’s expenditures on TAPS not yet prohibited.

### Article 21

Under Article 21 (reporting and exchange of information)^1^, Parties are required to report their implementation progress to the FCTC Secretariat, through the biennial submission of a report. In 2020, the Secretariat has enhanced the submission process with supporting materials, such as webinars^3^, step-by-step instructions^4^ and videos^5^ guiding each step of the reporting process. From the Parties’ individual reports, the Secretariat then prepares the ‘Global Progress Report on Implementation of the WHO Framework Convention on Tobacco Control’^6^, and submits it for consideration to the Conference of the Parties (the Convention’s governing body).

The report submitted by the Parties consists of a questionnaire, with sixteen questions referring to TAPS (thematic group C27) (Table 1). However, the TAPS section has a number of data entry issues^7-10^. The WHO FCTC Secretariat has reported^10^ that some of the Parties complete both the comprehensive ban questions (C271–C272) and the questions referring only to partial advertising restrictions (C273–C2711), which contradicts the step-by-step instructions, which suggest that the latter should be completed only when comprehensive TAPS bans are not present^4^. Additionally, amongst the Parties which have a comprehensive ban, a number give negative replies to some of the following ban sub-questions on media types covered by the ban (C272)^10^, which suggests that the Parties’ definition of ‘comprehensiveness’ varies^11^. Furthermore, some researchers^7-9^ are skeptical of the accuracy of the reports, as the data submitted are contingent on the Parties’ selective progress reporting^7^, the assessment is more nuanced than the data suggest^8,9^, and is not externally validated^8^. They also criticize the reported information as being discrepant with the existing situation, due to reporting of unrealistic policy changes in short time-periods or to under-recording of existing policies^7^.

**Table 1:** Simplified printed* version of the WHO-FCTC questionnaire section focused on Article 13 (tobacco advertising, promotion and sponsorship)

Question number	Question or instruction	Answer options
C27	Have you adopted and implemented, where appropriate, any legislative, executive, administrative or other measures or have you implemented, where appropriate, program?	
C271	Instituting a comprehensive ban on all tobacco advertising, promotion and sponsorship?	Yes/No
**If you answered ‘No’ to question 3.2.7.1, please proceed to question 3.2.7.3**		
C272	If you answered ‘Yes’ to question 3.2.7.1, does your ban cover: display and visibility of tobacco products at points of sales? the domestic Internet? the global Internet ? brand stretching and/or brand sharing? product placement as a means of advertising or promotion? the depiction of tobacco or tobacco use in entertainment media products? tobacco sponsorship of international events or activities and/or participants therein? contributions from tobacco companies to any other entity for ‘socially responsible causes’ and/or any other activities implemented under the umbrella of ‘corporate social responsibility’ by the tobacco industry? cross-border advertising, promotion and sponsorship originating from your territory? the same forms of cross-border advertising, promotion and sponsorship entering your territory for which domestic regulation apply?	Yes/No Yes/No Yes/No Yes/No Yes/No Yes/No Yes/No Yes/No Yes/No Yes/No
**Please proceed to question C2712**		
C273	If you answered ‘No’ to question 3.2.7.1, are you precluded by your constitution or constitutional principles from undertaking a comprehensive ban on tobacco advertising, promotion and sponsorship?	Yes/No
C274	–applying restrictions on all tobacco advertising, promotion and sponsorship?	Yes/No
C275	–applying restrictions on cross-border advertising, promotion and sponsorship originating from your territory with cross-border effects?	Yes/No
C276	–prohibiting those forms of tobacco advertising, promotion and sponsorship that promote a tobacco product by any means that are false, misleading, deceptive or likely to create an erroneous impression about its characteristics, health effects, hazards or emissions?	Yes/No
C277	–requiring that health or other appropriate warnings or messages accompany all tobacco advertising, promotion and sponsorship?	Yes/No
C278	–restricting the use of direct or indirect incentives that encourage the purchase of tobacco products by the public?	Yes/No
C279	–requiring the disclosure to relevant Government authorities of expenditures by the tobacco industry on advertising, promotion and sponsorship not yet prohibited?	Yes/No
C2710	–restricting tobacco advertising, promotion and sponsorship on: radio? television? print media? the domestic Internet? the global Internet? other media (please specify )?	Yes/No Yes/No Yes/No Yes/No Yes/No Yes/No
C2711	–restricting tobacco sponsorship of: international events and activities? participants therein?	Yes/No Yes/No
**Whether you answered ‘Yes’ or ‘No’ to question C271, are you:**		
C2712	–cooperating with other Parties in the development of technologies and other means necessary to facilitate the elimination of cross-border advertising?	Yes/No
C2713	–imposing penalties for cross-border advertising equal to those applicable to domestic advertising, promotion and sponsorship originating from your territory in accordance with national law?	Yes/No
C2714	Please provide a brief description of the progress made in implementing Article 13 (Tobacco advertising, promotion and sponsorship) in the past two years or since submission of your last report.	
C2715	Have you utilized the ‘Guidelines for implementation of Article 13 of the WHO FCTC’ when developing and implementing policies in this area?	Yes/No
C2716	If you answered ‘Yes’ to question 3.2.7.15, please provide details in the space below or refer to section F of the additional questionnaire available at this link. Response to this question or to the additional questionnaire is voluntary.	
C2717	If you have any other relevant information pertaining to but not covered in this section, please provide details in the space below.	

WHO FCTC: World Health Organization’s Framework Convention on Tobacco Control.

* In the digital version each of the questions appears on separate browser pages, one after the other. Hence, any inter-connection of the questions (e.g. from C273 to C2711) or instructions (like the one before question C2712), is not visible on any new opened browser page.

In an effort to improve reporting, other databases have been examined^7,8,12^, such as the WHO’s Report on the Global Tobacco Epidemic (MPOWER report)^13^, bespoke assessment tools have been created (e.g. Tobacco Control Scale, WHO MPOWER tobacco control score)^14,15^ or assessments with triangulated information have been conducted^9,16^. These alternatives have limitations too, such as the MPOWER report’s susceptiveness to both subjectivity and social desirability biases of the national experts who provide the scores^12,17^, and the subjectivity of the bespoke assessment tools during the scoring process^14^.

The FCTC’s reporting approach may not provide an accurate reflection of the actual policy landscape. Nevertheless, creating a new system for assessing TAPS policy adoption to the WHO FCTC standard would create an additional administrative burden for both the Convention Secretariat and the Parties. Identifying ways to improve the existing reporting tool may be more time and cost effective.

This pilot study evaluates the WHO FCTC questionnaire in terms of facilitating the Parties to fully capture the implementation of Article 13. To achieve this, we recruited and interviewed WHO FCTC national focal points (NFPs – individuals working as communication entry points between the Convention Secretariat and the Parties, designated also with the task to complete the WHO FCTC questionnaire on the Party’s behalf). Our study’s aim was to gain a deeper understanding of the completion process followed by the NFPs and of the challenges encountered during the completion of the WHO FCTC questionnaire section that captures the implementation of Article 13. The findings of this investigation could become valuable for the FCTC Secretariat, the representatives participating in the Conference of the Parties, the administrative personnel of the governmental tobacco control units, as well as the researchers using FCTC data on tobacco control implementation, and the tobacco control advocates seeking opportunities to support their government in monitoring the WHO FCTC implementation and its reporting.

## METHODS

### Study design and participants

We conducted qualitative one-to-one interviews with NFPs from low- and middle-income countries. Assessing the TAPS policy implementation in low- and middle-income countries (as those are encountering FCTC implementation challenges due to prioritization to other infectious diseases, the weaker legislative systems and the high reliance on international donor funds)^18-20^ and their reporting to the FCTC Secretariat, dictated purposive sampling of NFPs from this income group. The latter was identified with the use of the World Bank’s 2020 income country classification^21^, and the list of the FCTC Parties^2^.

In January 2021, the FCTC Secretariat facilitated our approach via an e-mail invitation to all NFPs from this group (n=81). The participation criteria were: being the individual who completed the FCTC questionnaire, having English-language competency, being employed as an NFP during the period of the interview, having access to a computer and Internet connection on the day of the interview, being available for an interview between January and March of 2021, and providing participation consent.

Due to low initial response (n=2), we prolonged the data collection period until June 2021, and sent a personalized invitation to 32 NFPs. These were proposed by the FCTC Secretariat due to their past close collaboration. As no further interest was expressed, we grouped the invitations of the 32 NFPs per WHO Region and we incentivized them by highlighting the opportunity to raise a voice for that Region. Another 8 NFPs then expressed their interest, but only 6 completed the consent form and scheduled an interview date. One NFP requested to conduct the interview in a written format, and this was granted. For the remaining 25 NFPs, 5 initially agreed to participate but later the communication was discontinued, one was relocated to another governmental department, one declined participation and 18 never followed up the communication attempts. We concluded the recruitment process in mid June 2021.

A total of 9 NFPs participated in the pilot study. The majority were males (67%), aged 31–54 years (mean: 44.7). They represented at least one WHO Region (two from each of Africa, Americas, and Europe, and one from each of Eastern Mediterranean, South-East Asia and Western Pacific) and their experience of completing the FCTC questionnaire ranged 2–11 years (mean: 5.6). Eight of the participants agreed to participate in a one-to-one interview of duration 30–60 minutes (average: 49) and one participant, due to language barriers and heavy daily workload, answered the interview guide in a written format.

### Data collection

The lead author carried out one-to-one, semi-structured interviews with all consenting participants. The interview guide (Supplementary file) was first pilot-tested with a former WHO regional employee with FCTC related responsibilities. The first section of the guide covered the participant’s role as an NFP (e.g. task responsibilities, training and instructions received), while the second was a cognitive interview evaluating the meanings and processes used by the participants to answer each of the FCTC questions. The participants were also provided with the opportunity to document any encountering challenges, both for individual questions and for the overall questionnaire, and to suggest improvements for future FCTC questionnaire versions. All interviews were conducted via Microsoft Teams, audio recorded via a digital dictaphone, transcribed, and then anonymized.

### Data analysis

A thematic framework analysis approach^22^ was used to analyze the data. The first two authors conducted the analysis through an iterative process of five main stages. The first stage of the analysis involved data familiarization by again listening to the audio-recorded interviews and reading all the interview transcripts, to obtain a broad overview of the participants’ responses. At the second stage, the first two authors compiled a list of topics emerging from the interviews (see analytical process in Supplementary file). Thereafter, they returned to the transcriptions, and independently made the identified topics more descriptive (see initial categories and themes in Supplementary file). The two authors met again to discuss them and to form the initial thematic framework (coding index). At the third stage, the two researchers independently applied the index to all transcripts using NVivo software, version 12. The researchers, following discussions, summarized the coded data under refined categories and synthesized the final themes (Supplementary file). This process set up the final analytical Framework (Table 2). At the fourth stage, the two authors independently applied the Framework to all transcripts to ensure its validity, and then discussed and resolved any discrepancies. Following this step, they charted the data in a matrix, with the main themes allocated to each row on the chart and each transcript assigned to a specific column (see full matrix in Supplementary file). The two authors used this matrix to identify the differences and similarities across transcripts and within themes. At the final stage, any identified patterns between the themes and categories were discussed amongst the first two authors, interpreted^22^ in the context of the reported criticism^7-9^ of the FCTC’s reporting process and then discussed in this manuscript. Throughout the analysis, the authors iteratively reflected on the original data and the previous analytical stages, to ensure the study participants’ views were represented and to reduce the possibility of misinterpretation. This pilot study was reported according to the consolidated criteria for reporting qualitative research (COREQ) (Supplementary file)^23^.

**Table 2:** Final analytical framework of the interviews conducted with national focal points from low- and middle-income countries in 2021, with examples of the identified themes and categories

Themes	Categories	Examples
**Use of different resources**	Answer basis	Law & constitution Previous submissions Monitoring data Combinations Experience
	Searching for guidance	FCTC Secretariat trainings and seminars Internal training Combinations Experience
	Involving others	Lone task Teamwork Consultancies Handling disagreements
**Compounding complexities**	Role struggles	Overwhelming workload Time consuming process Lack of knowledge & experience
	Questionnaire’s complexity	Lack of information Legal complexity and unclarity Language barriers Not knowing what is asked Unclear/unknown wording Restrictive answering Connectivity issues
**Supporting mechanisms**	External and internal facilitators	Legal clarity Questionnaire’s features Self-characteristics (experience, expertise, and knowledge) Collaborations (teamwork & consultancies)
**Recommendations for questionnaire and for those completing it**	Questionnaire development	Improve clarity Provide definitions Provide instructions Do piloting Improve structure Keep it the same
	Personal and organizational development	Gain legislative expertise Be legislation focused Attend FCTC’s trainings and workshops Involve others Time management and task prioritization

FCTC: Framework Convention on Tobacco Control.

## RESULTS

The analysis generated four themes: 1) use of different resources, 2) presence of compounding complexities, 3) use of supporting mechanisms employed for tackling the challenges, and 4) recommendations for refinements within the questionnaire and recommendations for those completing it (Table 2).

### Use of different resources

The TAPS section of the FCTC questionnaire includes three groups of questions. For the first two groups of questions (adoption and implementation of a comprehensive ban, and existence of TAPS restrictions), most participants based their answers on the content and language of the national legislation. Respectively for the latter group of questions (cooperation existence with other Parties, and utilization of the FCTC’s Guidelines), the participants drew from their own knowledge and experience when providing their answers. However, the resources used for the completion of the first two question groups was different amongst participants. Three participants (of the total nine) did not use the national legislation or when they did, they added supplementary approaches. Namely, as part of their answers’ justification, they used the TAPS compliance related information, or consulted the previous submitted reports and updated them according to any new adopted legislation or new TAPS surveillance data, whilst one participant used their own experience for completing the questions:

*‘… so I used my experience from filling similar questionnaires.’* (NFP, 1839)

The differences could be explained by the questionnaire completion instructions. The FCTC Secretariat provides relevant instructions on its website^4,5^, nevertheless participants did not mention using it. One participant avoided it due to the already existing administration burden:

*‘... I do not want to lie. I do not do that. Because I have a lot of things under…’* (NFP, 1239)

Instead, the participants chose other methods. They mentioned learning the process either directly from others (e.g. trained by the predecessor) or indirectly (e.g. own completion experience, attending FCTC’s knowledge exchange seminars) or by combining their own understanding of the completion process with their predecessors’ instructions.

Some study participants had support from a tobacco control team, while a few worked alone. All participants reported liaising with others (e.g. from other ministries, tobacco control committees, parliamentarians) for consultation purposes during the completion process; nevertheless the legislation content was always prioritized for answer justification over these consultations. In rare occasions of a disagreement during the questionnaire completion, the solution was found via team discussions, negotiations with policymakers or by training the individuals from the other agencies on Article 13:

*‘I … consult my team for providing a particular answer within the report. If there [are any] disagreements among the team for answering any particular question, I use [the] majority for resolving them ...’* (NFP, 1335)

### Compounding complexities

The completion process is burdened by the pre-existing struggles accompanying the NFPs’ role (e.g. overwhelming workload, limited human capacity and resources available, plethora of received information and requests). Thus, the participants identified the reporting process, including the data collection and verification, as time burdensome and the timeframes set by the FCTC Secretariat as not always clear. As an NFP highlighted, this task could become even more challenging for novice NFPs with insufficient experience:

*‘Many focal points … are changing [often]. If the new focal points … do not know what the Secretariat is, they do not know what the Convention is, they do not know what reporting is … it is impossible.’* (NFP, 614)

The complexities existing within the FCTC questionnaire do not ease the situation. During the interview, two questionnaire related barriers were identified. Firstly, the struggle to understand due to: a) language barriers, b) the question or legislation lack of clarity, and c) the unknown/unclear words existing within the questionnaire (e.g. definitions of the words ‘Parties’, ‘cooperation’, ‘brand stretching’). Whenever this struggle was encountered, participants provided their answers according to their own interpretation of the question or to the partial interpretation of the national legislation:

*‘Cross-border advertising… It is not clear to me. What does it mean? Is it for the internet? What is the difference?’* (NFP, 614)

The second issue was the challenge of fitting the available information from the local legislation on the questionnaire’s binary (yes/no) available options. Some participants found it difficult to answer some questions, due to: a) the absence of requested information, b) legislative changes during the questionnaire completion period, or c) the multiple ways to interpret the questions which was influenced by available information:

*‘So, I do not know in this context if I should answer “yes” or “no” ... what is the correct [answer].’* (NFP, 1839)

The participants overcame these barriers by using other non-health related legislations (in the case of a law’s absence) or guessing the most appropriate answer (yes/no) on the questionnaire based on the available information.

The two challenges are associated with the structure of the questionnaire. The current format of binary answers (yes/no) is restrictive. For example, whenever the legislation text or the survey’s question, or the results from the monitoring TAPS data, could be interpreted in multiple ways, the participants were forced to deploy their own interpretation about which option describes the legislative situation better:

*‘So if your process is looking on the one but not the other, it can be “yes” to one and “no” to the other … There is a lot of information.’* (NFP, 1827)

Similarly, the format of the first question (C272), which cross-examines the existence of a comprehensive TAPS ban, created a dilemma for some participants, since a negative answer prevents from informing the FCTC Secretariat about the existence of some of the TAPS bans:

*‘If I answer “no”, I will need to skip … the [section] C272 ... But within this block of questions there are questions that I can answer “yes”.’* (NFP, 1839)

The structure of the questionnaire also created some challenges in regard to the connection between some of the questions. This happened to the set of questions which asked about the existence of TAPS restrictions (questions C273–C2711). The first question (C273) asks about the existence of a constitution or constitutional principles precluding the adoption of comprehensive ban. However, all the following questions about the specific restrictions (C274–C2711) start with a dash and the restriction (e.g. ‘–applying restrictions on all TAPS?’):

*‘My question is if there is a link between question C273 and C274 … I do not know … this is a bit tricky…’* (NFP, 1839)

Hence, some participants answered these questions based on the existence of constitutional precludes and not the existence of a restriction, as the FCTC Secretariat requests^4^. Similar instances, of missing the connection from the initial question occurred in the C272 sub-questions – which inquire about the TAPS ban coverage; as well as in C2712 and C2713 – which refer to the existence of cooperation with other countries for tackling cross-border advertising and the penalties for cross-border advertisements. Missing this connection, the participants could misunderstand the questions’ true aim and provide answers which did not necessarily represent the questioned legislative environment.

### Supporting mechanisms

Despite these complexities, the informants also acknowledged mechanisms which made the questionnaire easier to be completed. The most significant mechanism was when a participant’s Party had integrated the FCTC’s Article 13 within the law and had the Article’s Guidelines translated and adapted to the local context, as reference to those documents made the completion process unchallenging. Other mechanisms identified were the questionnaire’s positive features (e.g. number of included questions), the NFPs’ collaborations with their teams or the other agencies, attendance at FCTC related workshops, and their own personal characteristics (e.g. experience, knowledge, expertise). However, the participants also acknowledged that not all supportive mechanisms apply for every NFP:

*‘It is not difficult to be answered [by] the person who has some experience in tobacco control. … For me [it is] not a problem … maybe for other people it is not [the] same.’* (NFP, 614)

### Recommendations for questionnaire and for those completing it

While some participants found the questionnaire ‘very simple and very straight forward’, many requested changes on the questionnaire’s clarity and structure. Participants who requested improvements on the questions’ clarity, struggled to suggest how this could be achieved. Only one participant suggested that the FCTC Secretariat needs to provide the definition of important words within each question, and completion instructions for the questions referring to corporate social responsibility, cross-border TAPS originating from Party’s territory, and banning the same forms of cross-border TAPS entering the Party’s territory for which domestic regulation apply (all sub-questions of C272). The same participant suggested that such definitions would help the NFPs to understand the questions and answer as the FCTC Secretariat requires. Furthermore, one participant expressed the opinion that the FCTC Secretariat should provide the questionnaire, its instructions and the FCTC website in more languages, while another recommended that whatever changes the FCTC Secretariat makes, they should pilot them first with the Parties and receive feedback about their appropriateness.

Three alterations to the questionnaire’s structure were proposed. Firstly, the removal of the restriction between questions C271 and C272 (‘If you answered “No” to question 3.2.7.1, please proceed to question 3.2.7.3.’), which provides a new block of questions only to the Parties that have a comprehensive ban on TAPS. Thus, everyone will have the opportunity to inform the FCTC Secretariat about the existing TAPS bans. Secondly, in question C272 (‘If you answered “Yes” to question 3.2.7.1, does your ban cover:’), it was suggested the repetition of its latter part in each of the following sub-questions which specify the TAPS types. Similarly, for the block of questions which refer to TAPS restrictions (C274–C2711), the participants suggested replacing any existing dashes with text clearly stating what information is required:

*‘My question is if there is a link between question C273 and C274. … if [in all sub-questions it asks whether] we have a restriction on tobacco advertising … [then it is] “yes”. But in the constitution, we do not have such a restriction!’* (NFP, 1839)

Lastly, some participants suggested that the questionnaire could be improved by providing explanations beyond the binary answer, such as through an open-ended section:

*‘There is a lot of information. And I think it should be some more responding options rather than just a simple “yes” or “no”.’* (NFP, 1839)

The study participants also suggested recommendations for those undertaking the NFP role. They suggested that every NFP should gain legislation expertise (local, on the Convention and on the Guidelines for Article 13) and be as close to the legislation’s content as possible during the completion process. Expanding expertise to other relevant legislation, outside of health policy, would also be beneficial. The participants also proposed that their colleagues attend small refresher trainings conducted by the FCTC Secretariat as these reduce time reading documents and also support knowledge exchange between the Parties. Furthermore, they recommended the involvement of others in the completion process, such as people from other governmental departments and the country’s WHO office:

*‘… my proposal for these countries is to use the power of the WHO [country] office … to … assist the national focal point to develop and complete the questions.’* (NFP, 1129)

## DISCUSSION

**Figure 1:**
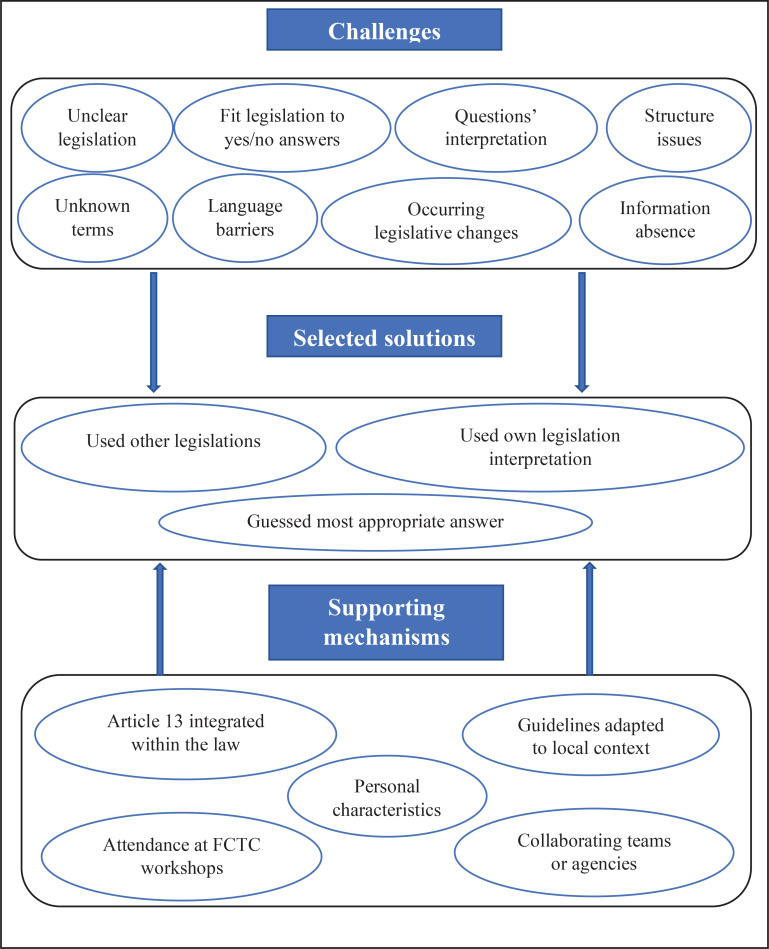
Challenges encountered during the completion of the FCTC questionnaire, and the solutions and supporting mechanisms deployed for overcoming them, according to national focal points interviewed in 2021

Our principal findings have shown some of the challenges such as different decision-making processes and other compounding complexities triggering the use of supportive mechanisms that are encountered by the NFPs during the completion of the WHO FCTC reporting questionnaire (Figure 1). Our pilot study participants provided recommendations to address these issues, both for the questionnaire itself and for those completing it. In the framework approach, the findings are then discussed in the context of the established literature and existing theoretical perspectives^22^. Below, we use previously reported criticisms^7-9^ of the WHO FCTC’s reporting processes to discuss how our findings relate and contribute to the understanding of reporting processes.

The FCTC Secretariat has stated^10^ that some Parties complete both the comprehensive ban questions (C271–C272) and the questions referred to advertising restrictions (C273–C2711). We did not encounter this in our pilot study. A possible explanation is that the interview process and the presence of the study researchers overviewing the questionnaire completion, might have increased the participants’ compliance when reading the questions and providing their answers. It should also be considered that several NFPs use the digital version of the questionnaire, which automatically bypasses the restriction related questions in case of a comprehensive ban reporting. Hence, such incidences can only occur in cases where NFPs use the questionnaire’s printed version, and due to their overwhelming workload may not pay sufficient attention during its completion. The recommendations to involve the WHO’s country office or the individuals consulting the NFPs in the completion process could minimize such occurrences.

The FCTC Secretariat also states^10^ that there are Parties with a comprehensive TAPS ban (C271) which did not actually cover all requirements (C272). We identify two potential explanations based on the study participant input. Firstly, it should be acknowledged that there are NFPs who considered the ‘comprehensiveness’ based on their overall interpretation of the legislation or based on the existence of the word ‘comprehensive’ within the legislation. Hence, the meaning of the word ‘comprehensive’ was not as tightly defined amongst the study participants as the WHO FCTC intends. Secondly, this incident may occur because, as one of our participants stated, some NFPs could feel uncomfortable for not reporting the explicit TAPS bans existing domestically, so they alter their answer in C271 by indicating that their Party is having a comprehensive ban while it is not. Within our data, there was a recommendation for the removal of the restriction between the questions C271 and C272. While this change could provide the opportunity for every Party to report any explicit bans, it would be unnecessarily questioning the existence of a comprehensive TAPS ban (C271). Adopting the participants recommendations in improving questionnaire clarity and structure, could help resolve this issue.

The NFPs have been criticized for selectively reporting existing legislation^7^, for providing nuanced interpretations, as opposed to yes/no answers^8,9^ and for providing answers that do not fully reflect the realities of the legislative environment^7^. These occurrences could be explained by the fact that the decision-making was different amongst the NFPs for some questions (e.g. questions around the existence of explicit TAPS bans), which incorporates other data (e.g. surveillance) than the legislation text; a discrepancy which is further complicated by issues related to the understanding of the question and fitting the information properly under the restrictive given options (yes/no answers). The latter options are forcing the NFPs to deploy their own interpretation of the question or the information (e.g. TAPS legislation) and under-reporting the existing policies respectively, which could lead to a reporting that is discrepant with the existing situation. The NFPs can minimize the effect of these complexities by deploying questionnaire completion facilitators and enhancing their capacity, by using the established or creating new collaborations, attending FCTC-related workshops to increase their knowledge and learn from other Parties, or by involving the WHO country office in the cross-examination of the data entries.

The differences in resources selection seems to be generated by the absence of standardized questionnaire completion instructions followed by all NFPs. Hence, the informant’s suggestion for including the definition and instructions for proper questions’ answering should be considered by the FCTC Secretariat. A within-questionnaire guidance could facilitate the novice NFPs with the completion and it would remove the challenge of altering the questions’ content entirely. The digital version of the FCTC questionnaire could easily be modified to underline the words commonly used within the FCTC and the TAPS field (e.g. Parties, brand-stretching, depiction); thus, when a mouse sensor overlaps these words then a dialogue box could depict their definition. Similarly, on the side of each question, the FCTC Secretariat could provide a detailed explanation of what is required from the NFPs to consider and what to avoid before answering the specific question. These additions could be burdensome for the FCTC Secretariat, however, as our pilot study found, the NFPs are not reading the step-by-step instructions or watching the video-assisting materials provided, and there is a need for a standardized approach for all NFPs during reporting to eliminate any questions’ ambiguity or own interpretations.

Lastly, the dashes within the questionnaire for replacing parts of the questions create the challenge of understanding the link to a parent question and they should be replaced. During our study, we noticed that the informants, after a couple of data entries, were forgetting the parent question and start assuming its content, often wrongly leading to data entry mistakes. For example, questions that referred to the existence of specific TAPS restrictions (C274–C2711) with the presence of dashes, could be easily read as questioning the existence of constitutional principles, thus precluding them from adoption, as stated in the parent question (C273). Since the questionnaire’s digital version compounds the issue, as every question with a dash appears on a new browser page, the FCTC Secretariat should change every dash existing within the questionnaire with the exact text that it replaces.

### Strengths and limitations

Our pilot study has some limitations. Due to the low participation rate, these data are not necessarily generalizable across all NFPs from low- and middle-income FCTC Parties. The presence of self-selection bias between our study participants should be acknowledged and thus the pilot study results need to be treated with the caution. Despite our efforts to for achieve high participation rates, these remained low. The participants were public officials, and hence we could not provide any incentives (e.g. vouchers) to increase participation. During the recruitment process of future investigations, anonymity and collection of non-sensitive data should be assured, clarification of own and field’s contribution, and that extensive expertise is not necessary for participation should be highlighted. Language barriers that prevent additional study participation could be mitigated by involving a multi-lingual research team.

The data’s generalizability to typical times should also be considered. The pilot study was conducted during the COVID-19 pandemic, which might have influenced the NFPs’ participation due to the increased workload and the specific challenges reported. The challenges described here, represent the opinions and recommendations of the people who were holding their country’s NFP position at the time of the study and for the FCTC questionnaire’s version circulated the same period. Hence, any challenges expressed here may differ from those that will be expressed from the participants’ successors who may have different professional background and expertise on the TAPS subject. Additionally, the format of the FCTC questionnaire can be updated at any moment, thus the challenges described here could be eliminated and new ones generated. Future studies could explore potential changes in the NFPs’ opinions over time or over updated versions of the WHO FCTC questionnaire.

**Figure 2:**
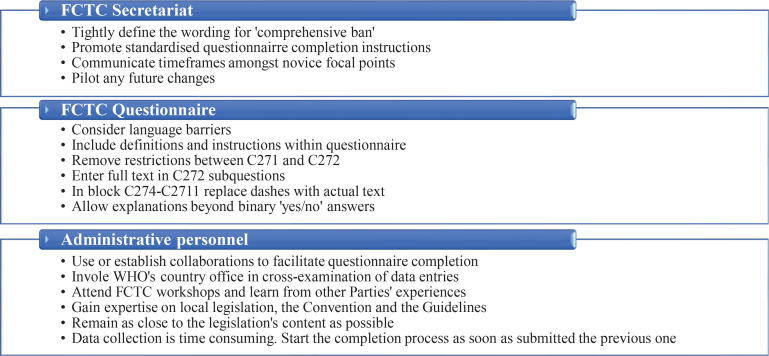
Summary of recommendations for the FCTC Secretariat, the questionnaire monitoring the implementation of the FCTC and the administrative personnel of the governmental tobacco control units, as provided by the national focal points that participated in interviews conducted in 2021

Despite these limitations, our pilot study also has some strengths for the general literature. To the authors’ best knowledge, this was the first ever investigation targeted to the specific specialists group. The small sample size allowed us to dedicate more time with each participant for every question existing within the section of the FCTC questionnaire that refers to Article 13. This helped the participants to explain their own meanings and processes, as well as to reveal struggles that were internalized until today. Our sample’s homogeneity revealed that the identified challenges were not associated with the participants’ specific WHO Region. In contrast, they seem to be influenced more by the personal interpretation of the questions, which was determined by the training received and the habits developed over the years with the FCTC questionnaire completion. The study participants provided an extensive list of recommendations (Figure 2). These should be acknowledged at FCTC level, as the outcomes from this questionnaire inform researchers and the Conference of Parties about the current state of the TAPS policy environment, are used as good practices exchange, and form the direction of the global tobacco control policy agenda. Our findings are also important for the national tobacco control advocates, as the challenges mentioned here create opportunities for initiating or further developing collaborations with the NFPs. This can be achieved by the advocates supporting the NFPs’ task, either by monitoring the accuracy of the Article 13 implementation reporting, or by facilitating novice NFPs with their training, informing them about the issues existing within the questionnaire, and supporting them with the proper interpretation of the questions.

## CONCLUSIONS

In this pilot study, we interviewed NFPs responsible for the completion of the FCTC questionnaire on behalf of their Party, and we documented the challenges encountered by them during the completion process, the potential causes of these issues, as well as the improvements which the FCTC Secretariat could make to the questionnaire. Addressing these issues could assist the NFPs to use a more unified data reporting approach and provide data that more accurately represent the TAPS policy environment of the Parties. Improving the data quality in such manner would help the FCTC Secretariat to be well-informed of what TAPS legislative gaps exist at an international level and identify how best to support full Party compliance with Article 13.

## Data Availability

The data supporting this research can be found in the Supplementary file.
