# Supplementary material for: Tobacco advertising, promotion, and sponsorship ban adoption: A pilot study of the reporting challenges faced by low- and middle-income countries
Source: Tob Induc Dis. 2023 Jan 23;21:10. doi: 10.18332/tid/155816 (PMC9869088; doi:10.18332/tid/155816)
Supplement: Supplementary file 1 [file TID-21-10-s1.pdf]

Supplementary File

Semi-structured interview guide (Ethics Code: EP 19-20_074)

Welcome:

- Self-introduction, present the aims of the study and the structure of the session
- Remind and explain: purpose of recording, right to withdraw at any stage of the interview, right to refuse answering any question making them feel uncomfortable.
- **<u>Remind the participant about audio preference</u>**
- **<u>Gain a verbal consent and start recording</u>**

Discussion:

1. Before we start the discussion about the FCTC self-report, I would really be interested in understanding more about your role. What is your **current profession and role,** and what is your professional **background**?
2. **Why** do believe the Ministry of Health approached you and assigned you **the role of the National Focal Point**? [probes: professional qualifications/expertise, member of the Ministry, local legislation knowledge]
3. How **long** have you been in this role?
4. Please **explain your role** in the completion of the report [Probes: volunteer assigned task? responsibility is part of a job position?] What **tasks** does this role involve?
5. Did you complete the report alone or did you work with a **team**? (if part of a team: Please **explain their role** in the completion of the report.)
6. What type of **instructions or training** did you receive, if any, to support you in completing the report? [probes: in-service training, pre-service education, on-the job, FCTC training & support, not trained] From **whom**? [probes: FCTC, MoH, just survey instructions] **When/How long ago**?
7. How would you **evaluate** these instructions/trainings now, after the completion of the survey? [Probes: adequate, need improvements]

Now, I am sharing with you the questions from the FCTC self-report which refer to Article 13. Please, **read aloud** the questions as like filling in the survey right now. While you are

reading them**, try to recall** and tell me which **resources, if any,** you used for answering each of the questions.

8. Thank you! Now, please, take another look at the survey. Which **questions** do you find particularly **easy or difficult**? **Why** particularly those? [Probes: questions which are straight-forward and do not require additional thinking OR questions which require to keep track of policy information, require external resources or consultation]
9. Do you believe these questions are also **challenging** for your colleagues **in other countries** or they are only difficult for **your country** setting? [probes: universal situation, individual country circumstances and challenges] **Why** do you believe so?
10. With whom would you **discuss** any difficult questions? [probes: other colleagues involved in completing the survey in other countries, colleagues within the team, MoH staff] **Why** with them? / **Why not**?
11. **How** did you eventually **address** these difficult questions?
12. (If NFP works with a team: Where there any **disagreements** among the team for answering any particular question? **How** did you **resolve** them?)
13. How could the difficult questions become **less challenging** in the future? [probes: more instructions, change their format, change wording]
14. Reflecting on your overall experience with the survey, **what** could be done to make your role and tasks **easier**? [probes: less questions, clearer FCTC instructions, more information from MoH, additional training, clearer definitions in the FCTC Guidelines] **In which parts** of the survey?

15. These are all the main questions I wanted to ask you. Is there anything you would like to add?
16. Could I contact you again if I have any further questions or need further clarifications?

Thank participants for their time. Remind them their right to withdraw their data within two weeks period.

Development process of the final themes from the interviews conducted with nine national focal points from low- and middle-income countries, in 2021.

<table>
<tr><th>Emerging topics from interviews</th><th>Initial themes</th><th>Initial categories</th><th>Refined categories</th><th>Final themes</th></tr>
<tr><td colspan="5">← Reflection on original data and analytical stages to ensure participants' accounts representation and to reduce the possibility of misinterpretation →</td></tr>
<tr><td rowspan="4">• Task management
• Answer basis during completion
• Barriers
• Facilitators
• Required improvements</td><td>Organisational processes used for questionnaire's completion</td><td>• Searching answers within main resources
• Choosing alternatives
• Involving & working with others</td><td>Underlying answer basis diversity
Opting instructions beyond the FCTC ones
Involving others</td><td rowspan="4">Use of different resources

Compounding complexities

Supporting mechanisms

Recommendations for questionnaire and for those completing it</td></tr>
<tr><td>Questionnaire's complexity & role's struggles</td><td>• Fighting with time and workload
• Knowledge gaps & experience
• Lack of information and legal complexity
• Struggle of understanding
• Structure complexity</td><td>Role struggles

Questionnaire's complexity</td></tr>
<tr><td>Existing and developing new facilitators</td><td>• Legal clarity
• Questionnaire's features
• Self-characteristics
• Collaborations</td><td>External and internal facilitators</td></tr>
<tr><td>FCTC questionnaire refinement and role-investment</td><td>• FCTC questionnaire design
• NFP's self-development
• Capacity building</td><td>Questionnaire developments

Personal and organisational development</td></tr>
</table>

FCTC: Framework Convention on Tobacco Control. NFP: national focal point.

Framework Matrix

Theme: Use of different resources

**Category: Answer basis**	**Informant**
It starts from "have adopted and implemented a TAPS policy and if this policy is comprehensive ban that covers all advertisements, all promotions, and all sponsorships". Well we do, that's checking the "yes".<br><br>Because we have a law that regulates the tobacco products like… with the… I do not know how to say this... not in English. It mentions that it comes in front of the tobacco baches… health warnings. Yeah, we have health warnings, we have… regulated by law in {named country} where these tobacco products can be or [not]… in stores. We have an age… age minimum to buy this kind of products. We have a law that denies… it is not "deny" the word… it does not allow tobacco industry to have sponsorship and advertising in radio, in… in all of that. Yeah, it's because we have a law to regulate that.<br><br>"display and visibility of tobacco products at points of sales?". Yes, as I mentioned, we have a law that regulates that this kind of products cannot be in the access of the people in stores. They only can buy something if they ask the people who sell them and they have to hide them behind the… access of the people who are buying them. They do not have to show… eh promotion… advertising.<br><br>"the domestic Internet". We do not have that. We do not regulate… not yet… domestic Internet… for advertising of tobacco industry. They do not do it that much… like cigarette… package… you do not see it that much. But we have seen vaping advertising in the social media… and we are trying to do changes in the law, because the attractive… is… might be to the young people. But right now, we do not have a domestic regulation.<br><br>"the global Internet". We do not have it. Not domestic and not global… we do not have that. Not internet. TV and other yeah, we do.<br><br>Oh, OK… we have that regulated… that they can not sponsor… like sport t-shirts from teams or they cannot give products like lighters or something. We do not permit that. We do not allow it.<br><br>"contributions from tobacco companies to any other entity for "socially responsible causes" and/or any other activities implemented under the umbrella of "corporate social responsibility" by the tobacco industry". It is not in the law, but they do not do this… not in {named country}. Tobacco industry, companies do not do some activities like sharing food and making… organizing events. This does not happen… but we do not have it in the law.<br><br>Let me see if I get this [right]… Yes, we have a regulation that does not allow to advertise anything. Like, if it's an international brand and they do it in their country, they cannot do it here. Because of the law.<br><br>OK, next one will be "Have you utilized the "Guidelines for implementation of Article 13 of the WHO FCTC" when developing and implementing policies in this area". Well, yes. We do usually base our decisions in the law about sponsorship, promotion of the companies in the… we are using the guidelines… of the FCTC from WHO... that [was] provided to us. Yes, that is the document that we have used. Most of it the lawyers… they used to bring everything… but yeah, we do. I do remember.	400

Because we have the law, so we base everything in the law… we do not guess, we do not try new things if we do not have it in the law. Because when we address to the companies, they demand us to have that in the law, otherwise they will not do it… the thing we ask. It has to be in the law, so we base everything in the law.<br><br>We usually organize the meeting and then we check every point to see if there is any change since the last [submitted report] one or if there is a new situation like when this law was written… you know… there was not so much of social media... and the internet was not that big… not that big in {named country}. In some situations that may present now might not incorporated in the question that we answered the last time for example. So we check every single question, and we discuss it and we talk about how is really the situation, not just in the paper but [also] in the streets... in the everyday life. If it is about advertising, have you seen any new advertising… are people not respecting… because we have a part of the institution that travels around the country to check if this… if the stores are not showing… I do not know how to say this… if the law has been enforced. And if we find some stores not respecting the law, we have some penalties. We have some penalties for the stores not enforcing the law. So we talk about that in the meeting and then we check… as a group, the questionnaire and then we answer. We have all completed and we agree. And we usually agree! And then we send it.	
OK. “have adopted and implemented a TAPS policy and if this policy is comprehensive ban that covers all advertisements, all promotions, and all sponsorships”. Yes, we have. We adopted and implemented legislative… we have a national law… for tobacco control… We ban of promotion, sponsorship… for tobacco. The real for… displaying… not for domestic Internet! We have some difficulty to have the internet… time to time we see in the internet promotion for tobacco.<br><br>The law forbids advertising and promotion for tobacco directly and indirectly. But for Internet we cannot… we have difficulty to survey this way of communication. In internet we see promotion for new tobacco product… we have promotion of new tobacco product in {name of country}. But the law is clear, it is… there is no authorisation of tobacco directly or indirectly by internet.<br><br>In the national tobacco control law, it is forbitten to have collaboration with… this program and the tobacco [industry]. It is [according to Article] 5.3… our law. OK? The corporation… the industry… time to time, they try to [go] against this law. But in our law… it is clear in the tobacco control law [to not cooperate].<br><br>It is the same answer because for cross-border it is very difficult for us to cover it… It is very difficult to cover it. In our law it is forbitten to do... to do advertising, promotion and sponsorship… in the law. It is right like it is, but in reality you have problem to… People do not observe cross-border [advertising].<br><br>We are having a comprehensive ban… the answer is yes. But in the real… this ban cover directly and indirectly promotion. OK? But in the real life, what I see… my colleague and I see… by the internet we have a violation of this law. But the domestic law… is clear. It is forbitten to do promotion for tobacco product by media. When we say media… media is net… classic media.<br><br>Yes {name of the country} has to collaborate and to facilitate for the elimination of cross-border advertising and tobacco traffic. As we have very traffic [of illegal tobacco] in our border. We have a collaboration with our neighbour countries like {named country}. We have to do it since… many years, to eliminate this traffic and promotion.<br><br>We have… it is very difficult. You have to do it in continuous program. Sometime the	614

{named neighbour country} focal point comes here and we share information and experience. We have them… For example, we help them to fight against traffic and promotion. They take our pictures and they put it in their tobacco project. It helps them to learn… to take our experience because {named NFP's country} has long experience for tobacco control. We have real problem with the traffic. We have also another one country that we collaborate…. {named another neighboured country}. But it is very difficult to follow… to do regularly meetings and to share information with all stakeholders of those countries.<br><br>We have imposed penalties… if you have a violation of the law. We have very heavy penalties for people in these cases.<br><br>In our law, because I am reading and I am answering according to the {name of the country} law… the tobacco control law…	
Because we have it on the tobacco control regulation. We have rules for that…. that regulation of {mentioned year of regulation}… [it] clearly mentioned that there is no promotion or advertising or sponsorship by tobacco industry in relation to tobacco or tobacco products.<br><br>"For the domestic Internet?" For that we have a team monitoring advertising through internet… as well. It is for domestic internet, I mean. The global internet, for time being we do not have any. So the answer will be "no".<br><br>The next question is "If you answered… display and visibility of tobacco products at points of sales?". At the moment we have no… any amendment yet, so tobacco can be seen from points of sale. In our new Act we are developing since {named year}… we added it in our new regulation. The Act is now under consideration we are waiting to be approved by the Parliament.<br><br>"product placement as a means of advertising or promotion". Yes, this is yes, because we have in our tobacco control regulation of {named year} as well.<br><br>We clearly state in the tobacco control regulation that there is no direct promotion… direct and indirect promotion for any tobacco product… from any tobacco companies… industries mainly by any means… through any sponsorships of event or CSR… so they shouldn't because indirectly would sell their products to… to their sponsorships… so that is what we put in the regulation… I think… yes.<br>INT: So you answer according to the legislation?<br>PA: Yes, we follow the legislation.<br><br>Ah… [No]… this is because we do not have any ban on cross-border [advertisement]… at the moment. In our regulation also doesn't clearly state that it should be ban… because… I think… is one of the… world trade organizations, so I do not think that we have… at the moment.<br><br>For this part there is none, because it clearly mentions in our regulation, so it can be clearly answered. Just for certain questions… like cross-border advertising, we are still on that yet… and also for… I think… the point of sales… because we are still lacking that part, so that is why there is "no".<br><br>We focus on both… I mean… specifically… in particular to the compliance data.<br><br>PA: "Have you utilized the..." Yes, we do.<br>INT: How?<br>PA: We… because… the FCTC… the WHO… we usually use to… as a reference in	702

which area we still lack… we will follow all the necessities and all the articles in the FCTC… the WHO FCTC articles.	
Based on the national tobacco control law, and any resolution of the national headquarters for tobacco control, I will fill [in] the whole section about the Article 13… questionnaire. Because for responding to any question, I have to refer to… refer the legal background… the legal resource.<br><br>Yeah, the section C273… "proceed to question 3.2.7.12."…" – applying restrictions on all tobacco<br>advertising, promotion and sponsorship?". Yes, we have applied. In all advertisement, promotion and sponsorship. Because again because of the comprehensive… the national guidelines.<br><br>In terms of cooperating with other Parties… our cooperation is in line with the development of the Guidelines at COP level. Because we are member of the Working Group. It is a mean of cooperation with other Parties for developing any Guidelines… in this issue. And also if any Parties have asked us to share our experience… we will provide to the Party our own experience. For example, our National Guidelines… we share our National Guidelines with the Parties who are looking for… seeking for the best practice and solution… these are the means of cooperation.<br><br>INT: What means of cooperation did you use?<br>PA: For the "technologies and any means necessary to facilitate the elimination of cross-border advertising"… there is no any cooperation. If there is… Actually no… We do not have any cooperation with any countries… or neighbour countries about the elimination of the cross-border advertising. Because there was no any… countries in this deal. To be cooperating for eliminating cross-border advertising. Because there is not any cross-border advertisement from all countries to other countries…. But we receive some examples of cross-border advertisement from the internet. The procedure of blocking the websites and the channels… virtual channels that containing images of tobacco advertising…cyber channels.	1129
Not really... No... instructions. They are giving us the instruction, it not changing year to year. But, we had no any formal training for this.<br>INT: So, whenever you are filling out the survey, it is like starting all over again the whole process?<br>PA2: It depends. We are looking at the previous report and if there are any changes in the legislation or we have a new, for example, survey [been] conducted and we have now new results… It depends… yeah.<br>For fortune, there are new things happening in {name of the FCTC Party} regarding tobacco, so we have also to fill in new information as well.<br><br>The only exception we have is eh… there are several questions here about {stated media type} here in the questionnaire as well… {stated media} is an exception. Unfortunately, there we do not have a direct law which regulates advertising on {stated media}. So we are missing advertisements on {stated media}.<br>INT: So from my understanding, you are actually balancing… Correct me if I am wrong… between the content of the law and your own experience of what you are seeing... outside, right? This is how you are deciding what to answer…<br>PA2: In general, it is based on the monitoring of the situation. We are doing this monitoring every day, in different places, including the advertisements. We have this information. It is not just our decision. It is based on objective… {participant asks in local language how it is called} ...<br>PA1: Yeah, objective outcomes. So we have percentages that there are no promotions for 99%... of tobacco.	1133

Ah yeah, ok I will go through it. So here, "display and visibility of tobacco products at points of sales?". Right now, so we had our legislation divided in two parts basically. One part initially banned the display outside… eh well, outside I mean [seen] from outside. So it should not be displayed and seen when you are walking from the street. And right now, ah… since [stated month] this year… eh… the second part of the legislation entered into force which bans display indoors as well. Like inside of the stores. And unfortunately, that law is, at the moment, violated because we lack the enforcement of the legislation… eh of the second part of the legislation. But the first part, which bans the display of the store, is well enforced.<br>INT: So eventually, how did you decide to answer this question. Because you said that half of it [the legislation] is implemented but the other half not. So, how did you choose what answer to provide?<br>PA1: you mean... yes or no? Yes, our legislation bans the visibility of the tobacco products... so we answered yes.<br><br>The "domestic internet". The domestic internet is unfortunately not been covered by the law. OK, the "global internet" is not regulated at the moment, so it will be "no" as well. "Brand stretching and/or brand sharing". That would be yes. Any type of sponsorship is banned... including the brand stretching and brand sharing. Then the "product placement as a means of advertising or promotion", this is also "yes" ... it is also banned. Eh... "the depiction of tobacco or tobacco use in entertainment media products" ... this is also banned, so this will also be "yes". But we see several cases of violations. Sometimes in printed media, where the pictures are printed for example of different events and [where the] people are smoking… It is not brand placement necessary, but by our… eh… legislation it is… eh… banned to display the act of smoking anywhere… as well… So, if someone is smoking on an event, you should not print that act on the magazine. Then, "tobacco sponsorship of international events or activities and/or participants therein". It is also banned and used to happen before {stated a year} quite often, but at the moment it is not happening… at least directly.<br><br>ah… year… "cooperating with other Parties in the development of technologies and other means necessary to facilitate the elimination of cross-border advertising". Eh... we do not necessary work in this direction… cross-border advertising, yet. Eh… We haven't actually had much cases about it either.<br>INT: Is it also mentioned in the legislation?<br>PA1: The cross-border advertising… not<br>INT: Cross-border cooperation… not cross-border advertising.<br>PA1: Ah… cooperation, yes! Not necessary about cross-border advertising.<br><br>Eh… as we mentioned, we… our legislation does not have the part where it regulates the cross-border advertising… eh… at the moment… this part we haven't regulated it yet.	
Yeah… the answer is "yes" because it is part of the comprehensive tobacco control act, we developed in {named year}.<br><br>Yes, it is because tobacco products are not visible. These [products] will be hidden when you request them, they will take them from there they are hidden, and they will sell them to you.<br><br>Yeah, it is written in the law. Of course if you display it, it means that you are advertising it.	1239
Yeah, everything is included in the new act. So the new act is very comprehensive…. It is everything included. All these answers here are "yes", we cover all. We go "yes" in all because they are part of the… I think the only one… we have two "no's", but all these are part of the comprehensive tobacco law act and regulation of {named year}.	

Whether we "are cooperating with other Parties in the development of technologies…" Eh… No. That one is not to my knowledge. Though we are cooperating like with {mentioned country}, but this one is on developing health warnings and also monitoring those graphical warnings. But in terms of advertisement, not yet.<br>INT: So you answer "no".<br>PA: …"are cooperating with other Parties in the development of technologies…"… in the development of technologies… "or other means to facilitate the elimination of cross-border advertising". No… we have not yet partner with someone. We wanted to partner with {named the previous country}, but not yet. On exercising tax we wanted to partner with the neighbour countries so we can at least have this. But on this one, it is not yet.<br><br>Yeah, we are having charges, but the charges are different.<br><br>Yeah, of course we do, because all the development of the policies, our acts, it is in line with it… these regulations, these Guidelines of the WHO FCTC.	
No, we have not adopted… well we have adopted, but not implemented. And when I say adopted, we have adopted the entire Treaty… Yes, all the aspects of the Treaty are part of our laws, but we have not implemented any aspects of the TAPS.<br><br>INT: So, for the following questions, you are replying negatively "no" in all points?<br>PA: Yeah. Now, when I was at SCOL although we will not answer this as "no", in what is required there…. Because we supposed to have the local or the national legislations and other measures and so on. But when persons do call, like for instance when the business sector regarding the TAPS, we do inform them that the [re]solution has been signed on to the Treaty and has actually passed the Treaty in our law. I think it was in 2007 the [re]solution passed the WHO FCTC Guidelines… they are part of our laws. So we were able to use them to prevent them from… you know… advertising and so on. But I think this because we [have not encountered] a true challenge from the tobacco industry. Somehow the message… when we say it is adopted in the law, it is passed in the law [and] they accept [it]… so we did not get a[ny] challenge from the tobacco industry regarding TAPS.<br><br>Not in our national… I am trying to explain that we do not have it in the Treaty and all the aspects of the Treaty that are part of our laws. But we do not have the specific laws and penalties and so on for this area. So, that is the gap… that we have.<br><br>OK. "If you answered "No" to question 3.2.7.1. are you precluded by your constitution or constitutional principles from undertaking a comprehensive ban on tobacco advertising, promotion and sponsorship?". No, no we are not precluded. Because the [re]solution has been passed into a law for WHO FCTC. First, we signed it and then we attributed… we made it a law. So, it is not our law books… it is the whole Treaty, all the Guidelines… the principles of the FCTC. But as I said before they do not have the particular instruments… the legal instruments in place to implement the ban on the TAPS. So, within our constitution it is definitely… it is constitutionally for us to do what we need to do to have it in the laws.<br><br>Yes, it is.<br>INT: So, have you use it? I am not sure that I understood if you have used it at local level, not regionally.<br>PA: It has been used at both levels. Both at regional level and at the local level. So my answer was… to 3.2.7.14 in terms of progress made, and my answer to the use of the Guidelines is "yes".	1827
In the legislation act, in {named country}, about advertising and sponsorship and so on… there is no mention about this. But, in the new law there is a… there is a restriction	1839

on tobacco use. In the law, there is a point that prohibits all forms of advertising or promotion of tobacco products, including direct or indirect advertising and sponsorship.<br><br>Yeah. In this document… all forms of advertising or promotion of tobacco products, including direct or indirect advertising and sponsorship… Then there is another article separated… about the prohibition of promotion of tobacco products, advertising and sale… there are a number of points… only two or three points about advertising.<br><br>For example, from my experience, because I was involved in many similar activities… filling in similar questionnaires, and also based on the WHO [FCTC] advice… that if I have such questions that are linked to each other… if I need to answer “no” to one of the questions to the block, then I have also to answer “no” to the rest questions. And that is why. Because I had the same situation in the block of questions C272, I had at least several that I had to answer “no”… so I used my experience from filling similar questionnaires.	
**Category: Searching for guidance**	
When I got in, the lawyers and the people who were already in the institution, they taught us everything that usually comes with all of our responsibilities in the institution, like [at] national and international [level].<br><br>We have a law to regulate tobacco in the country and… well mostly the lawyers explain everything to us and we have the legal documents that… we use to regulate the tobacco products in {named country}.	400
My experience? I have experience… I am medical professional on… in hospital and lead… coordinator of National Tobacco Control. I have the charge for reporting... since many years! …I have to share and to… implement the Tobacco Control in {name of country}. That means the tobacco advertising, promotion and sponsorship [which] is in our law. …and we have to implement this in {name of country}.	614
It is based on our understanding… on how to complete the survey and… that’s all. And also from my indirect teaching of my previous partners and colleagues.<br>INT: What do you mean by that?<br>PA: I mean, he or she will teach me on how to answer the questions… so this how we usually do it.<br>INT: So, when a new focal point take this position, you will have to train him or her accordingly?<br>PA: Yes	702
No… no… I just… it is a routine procedure and there is no need to be trained for… because every question of the report is clearly… actually written and as a… actually… explanation. So there is no need to have a training for the report. Yeah…	1129
Yeah… all started with the job training, because before I joined the unit or the program… the focal person was already there. The tobacco control aspect was already within the unit…. Before I joined the unit. So when I joined the unit I also continued with them, with no formal training even with the NCDs… even now I do not have a formal training on tobacco or NCDs in general. It is just in-the-job training and reading from the books and from workshops… experience and from here and there.<br><br>Oh no. Before being the focal person no… I did not receive any training. Nor on NCDs or on tobacco… I received no training. Only just in-the-job training as I said earlier on.<br><br>Eh… like WHO… When I say workshop [I mean] in illicit trade… organised workshop on country experience sort of… like one-two days training or workshops coming together… or sometimes Data to Action -we will call you and will invite you for a training where you will see our data and then the country experience… the country will also do a… country presentation based on the situation on the ground… what are some	1239

of the difficulties… the challenges… you also do your own presentation, so it is a form of a learning process. And the training I think... I also attending one training on TIM, that is the "Tobacco Industry and Monitoring" organised by {named a local university} in {named a different country}… that was just two weeks… that was in {named year} I think… I am not sure… but it was around that time. But basically, I attended a lot of workshops… a lot of seminars on tobacco, but not a tailored made training like a BSc program… or Master's program or PhD program… no. But I attending… I have been exposed to a lot of tobacco meetings where the countries there share their experience… yeah<br><br>I have not… no. I do not. Actually, I do not. Unless, like they… send an invitation… but for me personally to go to the FCTC website or to sites like these, or to read articles or to read other materials… no… I do not want to lie. I do not. I do not do that. Because I have a lot of things under… [if] you supposed to send an email to me… I will get it and then I will try and read… and I will try to find what you want and… exactly… then I will do… but like myself going to the website… the FCTC website or this sort of this… or training opportunities… no. I hardly do it. I cannot remember of doing it myself.	
I have not received instruction or training to support me in completing the FCTC report. However, I have been online meeting before publication of FCTC report. The convention secretarial described composition of FCTC report questionnaires and advice How to implementation the law in country under WHO-FCTC.	1335
I did a lot of reading. And also support from WHO… There have been a lot of trainings… courses, and opportunities to attend… and online webinars, as well as meetings in various countries when they allowed to travel and [thanks to the] support from the WHO FCTC.<br><br>Oh… do you mean the reporting questionnaire? Oh, no. That one was… discover as you go. And so if I needed any help, I would… I think I would be… I do not remember the person at the time… but in completing the questions, any top queries, I would send directly to the WHO FCTC Secretariat to the person responsible for administrating the questionnaire from that office.<br><br>Yes. We have the [re]solutions signed on to the Treaty, but went further and actually… took the Treaty to the Parliament, this is where the law makers have to pass it. So it went through that process, and it is now part of our law… the entire Treaty.	1827
No, no. Not any training, but… in sometimes the WHO FCTC conducted some trainings, seminars with all the Parties… {named WHO Region} Parties and we [ex]change information or experiences… yes, it [was] very good.	1839
**Category: Involving others**	
PA: Ok we have a legal department that we are usually consulting. In {named country} you do not need to be a lawyer to have a CO position. The CO is a dentist too right now… we are colleagues! We are friends! So, if we have something like legal… something… legal documents, we ask the lawyers of the institution. Usually, it is like… a door away.<br><br>We are always trying to do it in a group… with the lawyers… and the CO and… [myself]. Everything we talk about in the group re-union… like the middle room… and then we check everything is well written I guess…Like everything has to be checked, because everything comes in English or even in other languages.<br><br>The group that manages this topic is the two lawyers that handle this topic… and the CO and me. So, I wouldn't know anyone else to consult… [only] the two lawyers and the CO.	400

No we do not have... usually… like if we are not… Well, we do not disagree. We usually just respect the word that is in the law and everything that comes from the FCTC… but it doesn't happen… that we disagree for this kind of document… no, we do not.	
I answer to the questions of the FCTC… I share it with my team… If they have [any] information to [share with] me, I… to complete, you know… But I am the… I have the digital responsibility to answer to their questions.<br><br>No… No disagreements! It is an invitation. I call them to be with me, to hear about the interview, you know? To participate… if they have [any] information… to give [it to] us… to share it with us.<br><br>We do the training every time. It is our rule to… to… train our staff, OK? And focal point we have in {name of the country}, we have… some national… someone national committee for tobacco control. And time to time we have to train them about tobacco control law, survey… to how avoid tobacco… industry interfering… you know? To survey the… the promotion… for tobacco industry. We have to do it every time in our Program… and civil society also. We have to train civil society about how to work… and the NGOs about the tobacco industry activities.<br><br>They collect and monitoring… We have one staff… mostly we have one staff to share the information about industry interfering and transgressing our law. Every once we have one meeting where all the actors… civil society, national government actors and us, and we have to share… together our information and how to fight together the actions of the industry.<br><br>Before the National Tobacco Control Program started [the] only focal point for tobacco control in our Ministry [was] one person. Now we have a team. We have a team in the national program for tobacco control.	614
Usually, we will discuss in our team what areas that are in… especially in my own data so any information that they can go… can give to me, so that's how we collect the data… also from recent surveys, also national health mobility survey and all the policies we have here.<br><br>We have a team in charge for the screening and monitoring if there is any advertisement… especially in the Internet, the social media, and as well outside {outdoors} media… if there is any tobacco advertisement. As far as any information from the party if we receive any information. So we get this information and again we discuss in our team and develop the rules and with the enforcement offices [we discuss] what we can do. Usually, what we can do is… we have the laws to protect… to control… so… the one who did [any] mistakes or anything… so we can provide [proceed to] the relevant sanction.<br><br>We usually we have… if we have any disagreement, we are conducting a meeting like this, so that we can get an agreement… a consensus to the particular issue.<br><br>I am in charge… I mean as far as completing the questionnaires by the FCTC… so I am the WHO [representative] in charge in doing the direct… as far as answering all the questions in the additional questionnaire and the main questionnaire of the FCTC.<br><br>We usually we have… meetings mainly for the policy makers… especially in our sector, and also the sectors from the industry… those from the Custom Department, so we conduct the meetings quite regularly to discuss what is our lack and also about how to overcome the things we are lacking in our regulations.	702

I am the main responsible… I am the responsible for the… completing the report, the implementation report. I usually fill [in] separate sections of the report, but for other parts that need other agencies to [give a] response, I will send the report to other agencies, and I will coordinate with the other focal point of that agency to fill the separate… relevant part of the report. For example, if [the FCTC] are looking for some data from the Ministry of Industry about the production and everything related to the industry, I will send the report to the agency and I will ask them to send us formally… to complete the section. This is the process.<br><br>No, there are no disagreement… maybe there is some short of… how can I say that… misunderstanding. So, we have to train them… we have to explain them about the question… if they send any response to a question… to a specific question, and [if] I just feel that this answer does not match with the current situation at the country level, I approach the focal point at that agency and I ask them "why did you answer to this question in this term… so the main reason of this question was about that issue and you reported about the other issue". Then after receiving the formal letter… the report from the agency, it is my responsibility to verify the response from that agency. So, any case that I feel that the response is not correct I will approach the focal point from that agency and solve the problem.	1129
Yeah... Sometimes me or the Director General are mentioned as focal points. And sometimes {name of PA1}. It depends on different organisations.<br>INT: Just for me to clarify. Is {PA1} having an assisting role as national focal point?<br>PA2: Both of us are having the same role, I think. {PA1} is named the Tobacco Control person now. I am assisting [PA1]! (laugh).<br><br>Team task<br>INT: ...and how do you distribute the responsibilities?<br>PA2: So, there are several people in our group. For example, we are also having a lawyer who is working in the tobacco control direction. So, together with the lawyer we are also filling out the form, and then usually {PA1} is double checking whatever we have done and then [we are] sending it to the FCTC.<br><br>Between us?<br>INT: Yes… within the team… or with the FCTC if you had any further discussion with them.<br>PA2: The FCTC no… sometimes we have different answers in different questions, and we discuss it among us and… yeah… we come up with… eh… one answer.<br>INT: In order me to understand better the situation, could you give me an example about how you did you think different for a case and how did you resolve this disagreement?<br>PA2: Not exactly on this advertising part, but in general… for example how is the {mentioned legislation} is covered... {PA1} is saying 50% and I am saying 75% and we discuss… yeah.	1133
The tobacco laws… we have basically the tobacco control committee. This tobacco control committee is… The membership in this committee is 28 [members] according to the Tobacco Control Act… the provisions of the tobacco control act, section {named section}. They are from different Ministries and because I am the focal person for tobacco and [in] the Secretariat Conduct Committee the Director is the Chairperson to that committee. We have civil society organizations, we have NGOs, we have stakeholders from the Ministries, and we also have enforcers. We also identify three to four institutions that form part of the enforcement [team] of this Tobacco Control Act. All the laws we have, including the advertisement and sponsorship and those four… the public health officers… the Environmental health officers… those are the environmentalists…. the custom officers… those who work with the Revenue Department, and we also have the Police... they are also part of this. So, alongside we also form the tobacco monitoring team and the committee that I am also part of and	1239

other institutions like… the civil society they are also part of, the WO authority, the Ministry of Finance is also part of… the Ministry of Trade and the… Police Force, they are also part of it and the Law enforcement, and we also have the… biostatistics [team], they are also part of.	
integration data from other department, data validation across other department and summarize the country report.<br><br>I have the team to completion of the FCTC report including the public health professional and the Legal officer. They are main of responsibility of implementation law follow WHO-FCTC and the Article 13 of WHO-FCTC (Tobacco advertising, promotion and sponsorship) are part of {stated country} legislation. Consequently, they have responsibility of draft and enforcement law beneath WHO FCTC and they are main of completion of the FCTC report. Because the FCTC report is responsibility as a part of their current job position.<br><br>I work with my team and had consult with my team for providing a particular answer within the report. If there disagree among the team for answer any particular question, I use majority roles for resolve them and it [will] be accepted.<br><br>For the reporting process, preparation begins with the team and requesting relevant information from the other department. This is sent together with the WHO FCTC reporting questionnaire. The follow- up on data collection for WHO FCTC reporting is coordinated through the other department.	1335
But we do not have an established unit for tobacco control, where you have the persons for the various area roles. It is really one individual, you know.<br><br>Are you referring to the committees dealing with the cross-border? Yes, these challenges are discussed with other agencies.<br>INT: And how are these challenges been addressed?<br>PA: I do not know whether we were able to address [the issues]… But they have to do the questions… they have to help me with that. They need to go the process themselves… of getting the information and of giving feedback… to myself or to the committee.<br><br>We had to create a Committee, a multisectoral Committee and that has been very helpful. We call it the Tobacco Control Working Group. So we pulled together key people… we have pulled key people from the various agencies, as well as NGOs, like Cancer Society and those NGOs that have a concern for tobacco control. So TAPS… I am on the committee for TAPS… for the policies, the legislation and the other instruments and so on… for TAPS. So that is another responsibility that I have. So TAPS is spared among other agencies, so that is the solution we have for this. I just want to explain this set up about TAPS.	1827
No, I complete the report by myself [based] on the information that I collect	1839

Theme: Compounding complexities

**Category: Role struggles**	**Informant**
This Article 13? Usually it takes a whole morning… two or three hours to discuss in a group meeting.	400
The reporting for the [FCTC] Secretary… we need a little more time… because we need to translate and contact other focal points in our [other] departments. It takes time. It is difficult to have this information because they are having other activities… and to answer to our questions. It can take two weeks or more to have the information. But if we have old information, we can answer the survey in two or three days.	614

I think it is important to have building capacity. Many focal points you know… in countries are changing. If the new focal point that is working in the tobacco control program… they do not know what is the Secretariat, they do not know what is the Convention, they do not know what is reporting. And when the Secretariat sends them to do [the reporting]… it is impossible. They do not know what does it mean.	
Usually, it takes about… to answer this question… sometimes if the answer is yes, roughly about a day or two if the answer is still confusing. So we need to discuss this with the relevant agencies.<br><br>That's what I mentioned just now. We sometimes need to discuss this… we collect all the information first… what we need to discuss in detail, so we will have one or two day session. Mainly when this session… that's how we will discuss it and answer.	702
I think all of them are easy to answer. It depends on the actual… the current situation… the legal situation. If you have the resources… if you have the reference... the legal reference to answer the question is easy. But we do not have any legal… because it is a condition that may be different from one country to another.<br><br>The time depends on the process of reporting from the agencies. For completing this form it requires half an hour… but it needs to have other agencies' responses. So the procedure can take about one month… to get the answers from the agencies. Then, as soon as we receive the answers, I individually fill the answer in the report and in other sections of the instrument to submit to the WHO Secretariat.	1129
The only exception as we mentioned is {mentioned an advertising media not covered by the legislation} and we are working with different institutions and parties on trying to apply the same regulation for {mentioned the same media} advertising as well, but it is not an easy process you know… there are a lot of bureaucracies you have to go through. So you… eh… we are in the first stage of… eh… communications with different… eh… eh… Ministries involved.<br><br>…I mean it will be very hard for fill the whole survey for someone who does not know the subject and is not involved in this process. But whoever is, then is quite easy.	1133
But I do not have a formal training on NCDs or on tobacco control. …but from seminars, and [from] interactions with partners and exposure… so you know… things you have to be proactive if you are needed for a task… you pull [your sleeves] up and do whatever you can to meet up with these standards and meet with all the things you supposed to do. So that's it.<br><br>You find yourself in the job and you are exposed to it and you learn from your colleagues, from the books and when you go to these workshops… you learn from all colleagues from different countries, from shared experience so those are the most of the things I have been to… until now.<br><br>Well in the absence of what you want, you just take what you have! Yeah… I would like that... a training on it, rather than just two days – three days – one week training… is never enough. When you look on tobacco, it is a huge-huge area and it is a difficult area too… because tobacco control is not easy. You have a lot of challenges here and there and the resources are never available. This is basically in all {named the WHO} Region. The resources are very limited. And look the tobacco industry… they are so powerful. They can buy the whole country within a second. So you do your effort and in that you have to do so much research and some of those researches are difficult, because you just plan your things and then there is nothing there.<br><br>Yeah… my task is usually [being] the coordinator. Because the two… the research and	1239

the report… I will coordinate them and I will have people to work with. But these people… also some of them are not in the Ministry of Health, they are outside… in other Ministries. So getting them is difficult. And the capacity of the Ministry of Health in terms of research… it is not so much… So getting right people to work with you, you need to have also… something to at least motive them. So, it is challenging me and it is not very available. So, at least you support them… And myself, I have never been trained on [that]. So it is a big-big challenge. Sometimes I have been follow it on people… I used to do the research, to get them [what they need], but also thanking them for coming [to help] because [of] knowing that this is not part of [their job]… it is a challenge. So those are some other things. But we supposed to have people within the program, [that] would be different because it is part of your job and is your job, you have to do it whether [or not] you have talked what is part of your job. So if you are not trained for it, it is going to be difficult. You have to rely on people for some work. You can do what you can do. You have to limit [your expectations]… You cannot go for [collecting] the data. So it is a challenge.<br><br>Or just [by] printing the documents or sending them by email… you have other things to do… you just sometimes keep them in your emails and never download them.	
So, when the request comes out… eh… even that too. I, sometimes, am not sure… I have not fully understood when the next period is. So, sometimes it is when the questionnaire is sent out. Like, the last year I was not aware that last year was the year to report... So, I had not started collecting the data. Well… requesting the data from the various agencies. So, this is the other thing. We at the {Department}, we are trying to set up… how you call it… eh… regular… drug information network, so that we would be receiving this information on a regular basis, automatically when that agency submit [this information]. But while we are in the process of setting [this] up, at every point that we are to get this information, it is a long process… requesting from persons… sometimes we need to check… there are so many agencies that I have to physically call. Sometimes, I have this kind of networking with individuals, because you are [searching] for other routes sometimes. But you work together with the purpose of you are looking at. So last year was a rush. It was really difficult last year because the notice [by the FCTC] came very late and I had to spend quite a long time on it besides everything else… clear my desk and focus on just doing only this. And this, it is about two weeks to complete from the process of getting all the information, contacting the agencies first, getting the information from them and going back to the document now and be able to fill in questions [by] getting sure what the question is… what the question is asking, because sometimes in the tobacco we receive something that is not specifically… it is not in the format that the question is asking. So sometimes you have to make sure… and [there is] a lot of back and forth with the various agencies to understand. So, when you put this…. you know… what does this mean?… what does the data mean? To get some kind of interpretation of the data. So, I am answering the right questions in the form. Sometimes there is also a lot of… there was that aspect of getting the other reports. And those reports are in various formats. So we have the to write “yes” reports. We have STEPS reports. And those are the survey reports… and these different things… they are in different formats. You really need to be careful that you understand the reports. So there is a lot of reading, a lot of preparation that has to be into all of this... to prepare the reports.<br><br>OK, even before we start, I can tell you that this is one of the areas that is really-really very slow. And I should also mention that what we have to do in the absence of the tobacco control unit staffing…<br><br>Sometimes you fell… “oh Gosh!”… you know, you really want to do more, but I have learned that I am only one individual, and I can only do as that much. So for me, my strategy has been from 2017 to focus on one thing at the time because we have different	1827

other things to do. So from 2017 we have been implementing and adding to our MPOWER strategy… that is what we decided to focus on as well… we have been adding the other aspects one at the time… it has been helping.	
Yes, because despite [the fact] that I have the most recent information, I have [also] to check. I have to check again myself… to check again myself if there are any updates and ask my colleagues who may I consider [them] related to that area, if my answers are correct or no. So, I check my answers with my colleagues related to the area.	1839
**Category: Questionnaire's complexity**	
"brand stretching and/or brand sharing". This is something that I do not remember what [does it] mean.<br><br>OK, "product placement as a means of advertising or promotion". Yes, we have that regulated… they cannot use product placement, like to promote their brand nor the tobacco like cigarettes or vaping or nothing. That is regulated.<br>INT: In all media?<br>PA: …in TV, radio… well the law says internet too… but it is very difficult because we do not have that regulated. It is barely noticeable in the law… So, the [industries] tend to use that to take advantage of… to put some of… vaping promotion in the social media, as I told you. But not in the other media, like newspaper or anything… they cannot do that.<br>The next one will be "depiction of tobacco or tobacco use in entertainment media products". That would be a yes, it is regulated. They cannot use tobacco in the entertainment media like football events or sports in general. Or concerts… they cannot use that.<br>INT: What about the movies and films?<br>PA: Ah… the people cannot smoke… in no area… public area.<br>INT: I am referring to the actors who are smoking during a film. Is the depiction of smoking allowed?<br>PA: Well, that is not regulated by the law. So, if is anyone smoking or vaping in a movie is not regulated… in the TV too. It is not regulated. Like in there is a program and someone is smoking, we do not ban that, we only ban that industry cannot promote… [do] promotion or sponsorship. Is that ok? Does this answer to your question? OK…<br><br>"tobacco sponsorship of international events or activities and/or participants therein". I do not know… I do not know if I get this right. INT: This refers to… as you mentioned before football events, etc.<br>PA: Well, currently not international [ones]. It doesn't happen.INT: It does not happen, or is not regulated?<br>PA: We have it in the law, so they cannot…. In the country. We do not regulate [events] outside [of our] borders. I do not know if there is any sponsorship for the international events. I remember that I checked no… in that box.<br><br>Yes. "cooperating with other Parties in the development of technologies and other means necessary to facilitate the elimination of cross-border advertising". Yes, we do. But it is not regulated.<br>INT: Oh, no. This is only about cooperation. No! We do not have this. …As you can see I do not understand this. "Cooperating with other Parties…". We are… as a country, part of some research, like international… We are trying to do everything that WHO asks from us to increase the level of comprehensiveness of the [ban for the] FCTC… but I am not sure If this part of the question. Like with other… Parties? Like with other countries? Is that the question?<br><br>And the other would be [the question about] "cooperating with other Parties" … because I do not know which other would be involved. Like only the countries that are part of	400

the FCTC or something else. Because when we… if some country or someone look for us… like asks if we cooperate about tobacco, we always say "yes we do cooperate with other people".<br><br>But the question about the "cooperating with other Parties", I think it might be a little bit a problem for more countries than us. I think it is not clear. What do you mean about "Parties"? Like… the situation that I explained.<br><br>Maybe I do not manage some words, but I think it is good as it is. Except these about "cooperating with other Parties"… I think this is the only one that would be better to… I do not know how to say… fraction it… to have a better understanding of the question. That would be the only one. Because the next one is pretty clear. "imposing penalties for cross-border advertising"… yeah we do. But what do you mean about "Parties"? under development… yeah.<br><br>INT: So what could make it easier… more understandable?<br>PA: I do not know… I think it would be better to fraction it too… Three questions about if it's countries or non-governmental institutions, that works for these kinds of topics. Like cooperating with… whom? Because the rest of the question is OK. "development of technologies and other means"… I understand that, but what do you mean about "Parties". If I am reading this as an FCTC person, it [means] the other countries that are part of the FCTC. But only that or does include another type of organizations. That would be.<br><br>The [question about the] Guidelines is for us. Because of the language, the situation that we manage… the questionnaire as a group and not like one person.	
"Cross-border advertising, promotion…" It is not clear for me. What does it mean? Is it for the internet? What is the difference?<br><br>Yes. The reason is, because… I think it is… you are talking about promotion cross-country? Do you mean… promotion by internet? Now I can understand that it is like pictures and magazines.<br><br>The first difficulty is that the FCTC interface is in English. Sometimes, if you do not understand you have to translate the question before you answer it.<br><br>No it is no French version. The second difficulty is that it is not considering the survey. Sometimes they ask us to send them some documents many times. You are asking if we have any difficulties in our reporting. They also ask to document it. And sometimes we do not understand what do they mean.<br><br>I did not find the question difficult. I just want to be sure. What do you mean about the question. But it is not difficult for me. Our activity… our national program for implementation of the law we have to fight against industry interference... to fight against promotion [of] tobacco product[s], you know. It is not difficult for us because we have to do the reporting for the tobacco control and the… [FCTC] Secretariat. But you sent me the document in English. And my English is not [so good]… I use the French [version].<br>INT: Ah, yes there is also the French version.<br>PA: Yes. We have a translation… I am not English… fully.	614
For this particular section, I think the answer could be straight forward and easy if we have all the answers as "yes", or all the necessary… in our rules and regulation, but if there is none it would be quite be difficult and tough to say, because sometimes of the things we answer here is still under our discussion and also still under our provision to make it in our new regulations… and also that's why the answer is quite confusing. But	702

for certain things we should strictly stick in our regulation… we definitely… it is easy for me to understand.<br><br>Because there is no clear answer to the cross-border… that's why it's a bit difficult.<br><br>Because some of it is still under development… in the new Act… because it takes a long period for this… it is still not clear. Tobacco advertising [now] is different that the regulation we had in {named year}… the tobacco promotion and advertising [has been] evolved since then… [it is needed] a new regulation to ban or to control these products, I mean. We need to have a new clear regulation and Act to control this.<br><br>For time being… because as I mentioned… because the Act is still to be discussed in the Parliament... so, that's why the discussion is hanged still in there, because and we cannot proceed to collaborate more when we do not have the clear section from the Ministry to take part [in the discussion].<br><br>So far… so far we do not have any… eh… the answer is "no". We do not have any ban on the cross-border advertising yet.<br>INT: This question is about cooperating with other Parties in order to help you with eliminating cross-border advertising.<br>PA: So we do not have any cooperation… yet... At the moment.	
But it is not very clear… we need to disclose any tobacco industry's expenditure to the government authority, so we need to get the information from the tobacco industry. How can we approach the tobacco industry? Any expenditure of the tobacco industry… it needs more clarification for this question. This is the main question that I have.<br>INT: Does it require more clarification or does the Ministry of Industry needs to be more aware of the FCTC's Article 13? Because, if I am not mistaken this is a requirement by the FCTC…<br>PA: Yes, it is a requirement… but the issue of expenditures by the tobacco industries… mainly the expenditures are not tracked. But if you are track the expenditures of the tobacco advertisements, promotions and sponsorships we should directly prohibit them… from doing this issue. So, it is very important to respond to this question… Is it just to… actually to report that we have a legal infrastructure to not let the industry to extend for any advertisement, promotion and sponsorship. If they are looking for legal reference… the response is yes. But if they want to disclose any practice of the tobacco industry in this area, it is not easy to answer. We usually do not know how they act… about how they do the tobacco advertising. If we see them do this directly… we intervene. Because we have strong, complete, comprehensive national legal system on this issue. But in terms of any hidden intervention of tobacco industry it is not easy… traceable. So it is not easy to answer.<br><br>INT: But as I can see the question does not ask if you have or not have this requirement… but if your constitution prevents you from taking this measure? Please, go to C273, it says {reading the first part of the question}<br>PA: Yes, it is about the constitutional principles… so the answer is yes. But any example should be in a separate form… Yes.<br><br>For example, some of the questions of this questionnaire are not easy to answer directly. Because we do not have any straight-forward… any direct action for this question. We use other laws to answer this question.	1129
The same form of cross-border advertisement. Not the same form as entering our territory. That one is now. We have cross-border advertisement, but I think here we have the challenges with the fines. I guess the "same forms of cross-border advertising, promotion and sponsorship entering your territory for which domestic regulation apply". If it is [about] fine, it is not the same fine… but if it [asks] like whether we have this	1239

"cross-border advertisement" regulated, it is "yes".<br><br>INT: So the second part of this question is not so specific… for example about [including] the fine?<br>PA: Yeah. So I think the answer would be yes. Because we have the same… we have the cross-border advertisement stated in the tobacco control act.<br><br>"the same forms of cross-border advertising, promotion and sponsorship entering your territory for which domestic regulation apply"… I do not seem to I understand this one… I was thinking it is like the fines… can you explain this a little bit more?<br><br>…no comprehensive ban… "the same forms of cross-border advertising, promotion and sponsorship entering your territory for which domestic regulation apply"… What I know of, the ban is comprehensive and whether there is a cross-border [advertising ban] or within the country is all forms of advertisement is banned. Cross-border advertisement and the domestic advertisement they are all banned. So I think I can say "yes", it is banned… "Yes" it is banned.<br><br>I think these were the ones… eh… [C272] 13.7. Which state "the same forms of cross-border<br>advertising, promotion and sponsorship entering your territory for which domestic regulation apply". I do not seem to understand fully what the question is [asking about]. Apart from that one, I think all the rest are straight forward.<br><br>Are you asking if it is the same form?... cross-border… "Are the same form the same forms of cross-border advertising, promotion and sponsorship entering your territory for which domestic regulation apply"… Are the cross-border advertisement the same? I do not know whether they are asking… whether the cross-border advertisement and promotion and sponsorship "entering your territory for which domestic regulation apply"… I do not seem to fully understand… I do not know.<br><br>If I say "no", it means that it is not the same form of advertisement… because advertisement… cross-border… It is different. Sometimes the cross-border advertisement that we have and the advertisement we have domestically in the country is different… sometimes. Sometimes differs and sometimes is the same. So, it depends on the type of advertisement. This particular [question]… is a bit complicated. I do not know which… eh… the question… what specifically it means.<br>INT: Does it require a rephrasing?<br>PA: Yeah, it is not straight forward to me.<br><br>But here… to me the question is not straight forward. Whether it is asking about we have the same cross-border… like {named participant's country} and {named a neighbour country}. Do we have the same cross-border advertisement? Yes or no? Or [is it asking whether] the cross-border advertisement is it like the same with the domestic one? So, it is not much clear. It is just that the question is not clear. The domestic advertisement… we do experience domestic advertisement… cross-border domestic advertisement! That one is clear. And the penalties is different. If cross-border advertisement and… when you are inside, the domestic one is quite different. That one is quite clear. But here, the question to me is not that clear. I do not understand what the question is saying. This is why I do not want to come up with an answer… either "yes" or "no".<br><br>Because I said "no" here, but I am… because I was thinking if you are referring to whether we have these types or whether we do have the same types for cross-border advertisement. But… It seems that it states "the same forms of cross-border"… "the	

same forms of cross-border”. You know… this is a continuation. So, I do not know from where it links from! That is what I am looking at.… because it is a continuation. …“the same forms of cross-border advertising, promotion and sponsorship entering your territory for which domestic regulation apply”. So, meaning… it is a continuation from… maybe from question…?<br>INT: If you go to the previous page, it asks “If you answered “Yes” to question 3.2.7.1, does your ban cover:”. That is the origin of the question.<br>PA: What I have printed here, there is no {page} number.<br>INT: Then if you go before the questions with the bullet points.. {shared my screen with the participant and point the specific point}.<br>PA: Ah! … “does you ban cover…” “the same forms of cross-border advertising, promotion and sponsorship entering your territory for which domestic regulation apply?”. That one is “yes”. “Yes”! It covers the same.	
“applying restrictions on cross-border advertising, promotion and sponsorship originating from your territory with cross-border effects?”. This one… I am not so sure about. I remember having a discussion on this with the customs persons and we did not get any information for that. I am not that sure. So we would have to… I would be hesitant to answer to this because you would have to by directed also by our arrangements that we have… we are members of the {named a regional scheme} and we are also members of the {named another regional scheme}. So, that is a question that I am not able to say a “yes” or “no” regarding cross-border advertising.<br>INT: How do you decide what to answer? Is the answer coming from the committee you mentioned before? Or does the answer come from the customs agency?<br>PA: No. We are not having a determination of that. That is to be… we need more information.<br>INT: What about the next one?<br>PA: OK. The next one… “prohibiting those forms of tobacco” …Oh! But can I go to the first… to the previous one? We are looking for solutions as members of {mentioned a 3rd regional scheme}… with direct contact with these kind of standards. So we will… I can say “yes” in terms of {mentioned the 3rd regional scheme}, but in terms of international… I am not sure about that, but I can say “yes” in terms of {mentioned the 3rd regional scheme}. Because whatever it is decided, it will be the {mentioned the 3rd regional scheme} region.<br>INT: So, as it is the question right now, what answer do you provide? Because from my understanding, now you are having two types of interpretation.<br>PA: So it will be “yes” in terms of {mentioned the 3rd regional scheme}. The question does not give you that… You see it is either a “yes” or “no”. I have to interpret the… My interpretation would be “yes” but limited to {mentioned the 3rd regional scheme}.<br><br>“prohibiting those forms of tobacco advertising, promotion and sponsorship that promote a tobacco product by any means that are false, misleading, deceptive or likely to create an erroneous impression about its characteristics, health effects, hazards or emissions?”. Yeah, I think we do that. Yes, that is a “yes”. Based on, you know, whatever we have already said about the FCTC. That is constitutional for us to do that.<br><br>Yes, I was explaining… we as constitutional, it is part of our law…. The FCTC Guidelines and the principals. We have to take anybody to task. If we have to implement… if we have to apply any further things, there is anything specific to those areas in our laws, because these specific legislations have to be drafted and have to be passed. And I do not know if that helps… if it is an explanation.<br>INT: It helps, thank you. Please proceed with the next one.<br>PA: “requiring that health or other appropriate warnings or messages accompany all tobacco advertising, promotion and sponsorship?”. I would say “yes”, we can, our constitution does allow that.<br>“restricting the use of direct or indirect incentives that encourage the purchase of	1827

tobacco products by the public?". I would say "yes". We do not have the instruments to do it, but we are allowed constitutionally to create these legal instruments to control them.<br>"requiring the disclosure to relevant Government authorities of expenditures by the tobacco industry on advertising, promotion and sponsorship not yet prohibited?". It is not been done, but yes it can be done. There is anything in our laws that would restrict that… As far as I know…<br>So, I think that's it for this section.<br><br>INT: And what about the restrictions on sponsorships of "international events and activities?" or "participants therein?"<br>PA: Eh… I am not sure about international events… I am not sure… If we can restrict for international events. I do not know what to answer here.<br>INT: So what answer do you usually provide here?<br>PA: …"restricting tobacco sponsorship of"… "international…" I would say… the answer is "no". Because that is going to be… that is going to be under [re]solutions… and influencers admissions. I am not sure that we can restrict international events. Like for instance… I do not know if this is a good example, but I am thinking of the football league or something like… I am thinking it as an international event… unless we loose [the right of] participating and sending representatives… we will not be able to control that, but if we are the ones participating in that [events], obviously the answer is "yes". So for participants our nationals are participating, so "yes", but for internationals events we have no control of, so the answer is "no".<br><br>And I would say the answer is… let me see what the question is… is if we are restricted or if we are not restricted… We can actually do this. Actually, this is one of the things we are working on… that is the approach we are taking on.<br>INT: And what type of cooperation are you doing?<br>PA: Ah… solutions working on the Committee, that is actually going to be advice, standards… advice in policy basically and standards and etc… the standards would be mandatory. Standards or laws that would govern the TAPS and I think [Ministry of] Customs was trying to make those laws compliant… as compliant as possible with the FCTC.<br>INT: If I am not mistaken, this question refers to the development of technologies.<br>PA: Hm… it is "development of technologies and other means". I am interpreting it in a broad way. As we do not have any of those standards existing. So when is that [in place], then we will be able to see… what is that we need. Is it some kind of special technology to be able to monitor or to be able to implement, so anything….<br><br>From the ones that we just looked through? Eh… the ones that talked about cross-border [advertisement] and international one… that ones are quite difficult.<br>INT: Why those ones?<br>PA: We do not have that information… I do not have this information available as… what is going to be done, what can be done… that goes beyond my knowledge and beyond my control. So, this is where we have to…<br><br>think I noticed there… where there are just "yes" or "no" answers… some things are not so simple… And a lot of questions are compounding. So they are asking for… for instance C2712, that's "cooperating with other Parties in the development of technologies and other means". So if your process is looking on the one but not the other, it can be "yes" to one and "no" to the other. And some other questions are compounding. There is a lot of information.	
This question is a bit complicated. If I answer "no", I would need to skip another way of questions… the [section] C272, because it prompts the C272. But within this block of questions there questions that I can answer "yes"…. Positively. That's why I think the	1839

question 3.2.7.1 should be reformulated. I do not know… do you understand me? In the C273 [block of questions], there is even more complexity. “if you answered “no””…<br>INT: Could you please explain to me why is it more complicated this one?<br>PA: Just one moment… OK… In C273 my answer is “no”, yes? Totally! And then the next questions they are looking as separate [questions]… or [they are] not? Just [to give you] an example. For example, if I give a negative answer to the question C273…. [and then go to] “applying restrictions on all tobacco advertising, promotion and sponsorship?”, [then] my answer is “yes”! I answer positively to the next question. I do not understand the questions. I answer to the next question from the point that… “is there a restriction”? [then my answer is] “No”. I think, I probably have to answer “No” in order to be [in line] with the previous question. So how can I understand all these questions… connected to each other? …or should they be answered separately? I mean, is C274 connect to C273? Or not? Should I understand C274 means constitution restricts applying restriction on all tobacco advertising, promotion and sponsorship? Should I consider this question connected to the [“precluded by” the] constitution or not? Then my answer should be “no”! …but I answered “yes”. My answer depends on how I consider the question with the relation to the [“precluded by” the] constitution or without the relation to the [“precluded by” the] constitution. Probably, I answered incorrectly… I answered “yes” because we have such restrictions. But probably I had to answer “no”, because there are not such restrictions in the constitution. Probably in the C273, there [should] not to be a question side or… In the question C271, there is a branch for one question, and depending on answer to the question you skip, or you proceed to the next question… But here, in regards the question C273, it is a bit vague. Are they connected to each other or not. Should we consider them linked to each other or not. So, that is why it is a bit complicated.<br><br>Another [issue] in question C271, also I think there is some kind of… difficulties. Because in some situations, even if I answer “no”, there are questions [in the following C272 section] that I can answer “yes”.<br><br>I do not remember exactly, but there are some points to which we have put the response “yes”. And if you would not know about these [sub-questions about the ban coverage]… in the report you cannot reflect that because, as I said, you have to skip the answers [of the question C272].<br>INT: OK…<br>PA: Regarding [the] second point, it should be better if… My question is if there is a link between question C273 and C274. For example if we have a restriction on tobacco advertising that is why I answered “yes”. But in the constitution, we do not have such a restriction! I do not know… this is a bit tricky…<br><br>So actually, we are discussing these topics while we meet with our colleagues in the different WHO or FCTC events. So, I do not know in this context if I should answer “yes” or “no”... what is the correct [answer]. We do not have specific events related to that, but during the WHO FCTC events… but maybe there are such events, because according to the new legislation that we have in a higher level… higher governmental levels… there are such activities of which I am not aware of.	

Theme: Supporting mechanisms

Category: External and internal facilitators	Informant
Well this is part from a larger document and I think the size is ok. It covers a lot of a good information.	400
Now, I cannot explain it to you. When we were writing our law, we used it. OK? We used the recommendation, because in our law promotion for tobacco [products] forbitten. We used these guidelines for tobacco regulation.<br><br>I did not find difficult for many reasons. I am in the tobacco [control field] for many years, because since I am working in the tobacco control, I am specialized in tobacco epidemiology. I am working for FCTC… at COP… and {named an identifiable position} and I work many years for helping some countries to have law and fight tobacco [industry] and how to implement the Protocol [for Illicit Trade]. I am working… I am {named an identifiable held position}…I have no difficulty to this. It is my expertise.<br><br>For me it is not difficult to understand it and answer it. It is a good questionnaire, it has logic, like... [it is] right. The questions, I think, are clear. It is not difficult to be answered [by] the focal point or the person who has some experience in tobacco control. It is easy for the people, I think, to answer the questionnaire.<br><br>For me [it is] no problem because I know what does it mean “Guidelines for implementation”… maybe for other people it is not [the] same.	614
INT: Please remind me, what is the background of the people participating in this meeting?<br>PA: The custom, the ministry of trade… ministry local trade, ministry of customs and finance, but mainly the custom department.<br>INT: Do you have any lawyers participating who can help you with some parts of the law?<br>PA: Sometimes we are involving lawyers in these sessions. Lets [say] we have something that we really need to clarify… so they [give] us their opinion.	702
And as you know we submitted, we have ratified the Guidelines… the National Guidelines on Tobacco Advertisement, Promotion and Sponsorship. These Guidelines is about definition of the tobacco advertisement… comprehensively defined… Because according to the Article 3 of the National Tobacco Control law any advertisement of tobacco is forbitten, but there was no actually… there was not any definition of advertisement… So we decided to develop a Guideline… a National Guidelines… what is advertisement… what are the symbol of advertisement… In the judicial system, it is very important that they know… if we refer any violation to the judicial system the judge should refer to the legal [document]… for example if they say that promotion at point-of-sale is banned, the judge should know if he puts any instrument of tobacco in front of the public’s view, [whether] is it legally banned or is it legally [a] violation. So, when we drafted the Guidelines it is very clear for the judicial system to judge and decide about the criminal or about the violence. So, in completing this form, I just refer to the Guidelines and also to the National Tobacco Control Law.<br><br>Exactly! Yeah. It has been developed with representatives of relevant agencies. It took more than six months to [be] developed. Yes. We invited all of the representatives of the relevant agencies… mainly the ones who are members of the national tobacco control headquarters. For example, there were from the Ministry of Education, Ministry of Culture, and representatives from the Police Commander, representatives from the NGOs and also from the {named acronyms of organization}… yeah… the national broadcasting organization. So, they [were] invited to the Committee and to discuss about the issue of the Guidelines and then completed the draft. So they submitted it to the	1129

headquarters. Then they got the approval of the headquarter and also the President in {named year}. These Guidelines have been ratified by the President in {named year}.<br>INT: These Guidelines, where they formulated on the baseline of the FCTC's Guidelines for the Implementation of Article 13?<br>PA: Yes, the main text is inspired by the FCTC's Guidelines. Yes.<br><br>So, regarding adopting a comprehensive ban on tobacco advertising and sponsorship the response was "yes"… and this is because of the comprehensiveness of the Guidelines… the National Guidelines. Because it is comprehensive… it meets every issue related to the comprehensive ban of advertisement, promotion and sponsorship. So, the response was "yes"… it is still "yes"…<br><br>So the national tobacco control focal point should be very [in] alert and very informed about the current situation of the country… any part of the Convention… and have enough experience on this issue… and have a good monitoring system at country level. Because for example we through the University of Medical Sciences… the Environmental Health sections throughout the country and ask them to report any violation on tobacco advertising and any case… because besides the formal system we have… we have a hotline… we have established a hotline… a compliance public hotline that people report any violation on this issue. So, we have announced this hotline to the country… and ask people to report any violation for any article of the national tobacco control law… for example advertisement, any other issues… smoking at the public place, another issue… so we then receive suck kind of compliance report from the public, we match this report with the report of the agency and any actual difference between the reports will be followed-up to solve the problem. So the verification is very important…<br><br>The number of the questions is enough… it is good, because we need to meet every part of the Convention… and the Article 13.	
..eh… yes.. So… eh… in order to develop this legislation… whatever we are… have at that moment it was developed in line with the WHO FCTC. So we had all the guidelines and the articles even translated to {mentioned local language} and we worked through this document in order to develop our legislation.<br><br>PA2: No, no… it is ok. Eh… not too easy, not too difficult.<br>INT: What about its length?<br>PA2: It is ok too.<br>{…}<br>PA2: All questions are quite well understandable.<br>PA1: Yes… and relevant. So somebody who is working in this field, should have an... eh…<br>PA2: …good understanding<br>PA1: …of all the points provided here. So it is not like somebody needs to highlight one specific point. The focal point should know all of it.	1133
And we also observe because we leave with them, we have the committees, we have friends, and we have networks also… root groups, we have WhatsApp and other meetings. So any form of advertisement or sponsorship that is going on, we are [getting] alerted.<br><br>No… it is simple… We just read the Article 13, the Guidelines, because we have the Guidelines at hand… we read the Guidelines and we make sure that whatever we are doing is in line with the Guidelines… the formulation under. So that's how we usually do it. The advertisement, the ban of advertisement… the stated ban on advertisement… we use the Guide[lines] of the FCTC protocols… and also the policy. When we develop the policy we have these in front of us… we read and see… make sure that whatever we	1239

adopt as a policy or as regulation or as an act… it should be in conformity with what the Guidelines state.<br>INT: What is your opinion about the Guidelines? As a document… is it helpful or is it confusing in any parts?<br>PA: To me, I think it is very helpful. It is so comprehensive…. itself… it is so comprehensive.<br><br>No… the others are all fine. They are all straight forward.<br><br>The questionnaire? Yeah. I think, for me, the questionnaire is very simple and very straight forward.	
All of questions have an intermediate level. Because we are understanding of Article 13 (Tobacco advertising, promotion and sponsorship) and we adopted and implementation on our {stated tobacco control act}.<br><br>I would discuss any difficult questions with the team. Because we are work together for implementation of Article 13 of the WHO framework convention on tobacco control.<br><br>I answer questions from my experience that I as focal point of Office of tobacco products control committee and implementation of Article 13 of the WHO framework convention on tobacco control. So, all of questions are not difficult question for me.	1335
Well, I mean… [in] every meeting and every presentation you get the overall… the general explanation of the Treaty, the various aspects of the Treaty.<br><br>So that training, I remember, really gave me very good insights of the… great understanding of what tobacco control is all about and what the MPOWER package was all about, and the Treaty itself as well. Also where we are in terms of the various kind of countries for submission in the last four months on the tobacco control and what our goals should be, and why it is so important on the health impacts and so on. So, that kinda made me really to concern… and I was able to understand what… to have a better understanding of what we need to do and the direction… to go forward. And also being a member of the NCD Committee, Noncommunicable Diseases Committee of the Ministry of Health with more focus on tobacco control aspects… that also helped me to understand the great role of tobacco exposure and the importance of preventing premature deaths.<br><br>OK. So, as I have said {named country} is not only participating on the {named a regional TAPS committee}… we are… I think we are also the Secretariat. A committee has been set up by local persons and this is been led by {named a regional committee on TAPS}. So as the tobacco control focal point I have a representation. I am the representative on… I am a member of that committee. I think there is also {named country}… is a member of the regional committee. That has to do as a Region in implementing the TAPS… the ban on TAPS.<br><br>So, this is where we have to… I have to partner with other agencies, for instance the Ministry of Foreign Affairs, with the Attorney General and all of these agencies that deal with the laws… the international laws, what treaties and solutions [we are] part of… or other trade treaties that maybe restrict it.<br><br>Well, these will be the ones which are asking what is happening, what has been administrated for the last few years and what is happening locally. We as tobacco control focal points we have been able to have a representation of the various committees that are actually working and receiving information from the other agencies… to be involved in the operations related to tobacco. That works.	1827

I mean we have responsibilities for reporting and for implementing. So what I see is that we have quite a lot of support from the FCTC… Even sometimes I feel that I am bombarded with all of the… you know… the information that comes from them, and I do try to attend as much of the training as possible. You see all the activities that are been done, the report on the global situation… what other countries are doing and the advances over here and advances over there... Sometimes you fell… "oh Gosh!"… you know, you really want to do more<br><br>Eh… the TAPS section… it didn't really take that much time you know, because it is not asking to include any data that you need to find of what is happening. So this section is pretty short. You are just making sure that you read the question and understand what is asking. So, that didn't take that much time at all.<br><br>No… for just the TAPS? That didn't take me more than maybe… an hour or two? Yeah… It is because, also the [re]solutions are not so advanced in this area. So most of the questions do not really… there is not much happening as yet. So, it was pretty straight forward… we are not that advanced in these areas… there was not much thinking and checking and so on to be done.<br><br>We had to create a Committee, a multisectoral Committee and that has been very helpful. We call it the Tobacco Control Working Group. So we pulled together key people… we have pulled key people from the various agencies, as well as NGOs, like Cancer Society and those NGOs that have a concern for tobacco control. So TAPS… I am on the committee for TAPS… for the policies, the legislation and the other instruments and so on… for TAPS. So that is another responsibility that I have. So TAPS is spared among other agencies, so that is the solution we have for this. I just want to explain this set up about TAPS.<br><br>I have to partner with other agencies, for instance the Ministry of Foreign Affairs, with the Attorney General and all of these agencies that deal with the laws… the international laws, what treaties and solutions [we are] part of… or other trade treaties that maybe restrict it.<br><br>Well, these will be the ones which are asking what is happening, what has been administrated for the last few years and what is happening locally. We as tobacco control focal points we have been able to have a representation of the various committees that are actually working and receiving information from the other agencies… to be involved in the operations related to tobacco. That works.	
But I would like to mention that those events are happening, more or less, on a constant base. And that was useful for every participant… those events. Because [of] the exchange of the experience… So, before I had to start the reporting process, I had this kind of meetings [been] invited [at]. And these meeting were connected with other similar topics [like] NCDs, tobacco… They were very useful, and to my opinion the whole process of operation of tobacco use is one of the most advanced in the public health area. Considering the other public health topics, the intensity of work in the area of tobacco prevention and tobacco control it makes us the possibility to advance it… considering the other public health topics. And this brings positive results.<br>INT: So, if you had a new person taking over the national focal point position, would you recommend this person to join these meetings?<br>PA: Yes, definitely.<br><br>For your information, in {named year when participant took over the national focal point position} within the Bloomberg [Philanthropies] project about tobacco [control] grants, they gave us for a web-portal. In {named year}, we updated this portal and… at this time the portal is… [contains] resources with some legislation acts. So I update the	1839

content of this portal when the legislation is changed…. when there are changes in the legislations related to tobacco… those changes are updated… constantly on the web-portal that I mentioned. You can see in this portal… and this can help me to fill in the related reports… with updating, with some changing and so on. This process is negotiated with some policy makers, like in the Parliament. Those are negotiated with Parliamentarians related to the tobacco control activities and… involve them in our public health activities related to the tobacco control. It helps me fill in [the FCTC report] more accurately.<br><br>We have contacts with the Parliament. We have for example [persons-]links to the commissions of the parliament, so they help us to connect to the correct people in the Parliament for the most recent information.<br><br>Yeah, I would like just to mention that because we are having many activities in the country regarding to the tobacco control, we have a platform to develop [everything] faster. As we say, "the only ones who do not mistakes, are those who do nothing".<br><br>My answer is "no". So actually, we are discussing these topics while we meet with our colleagues in the different WHO or FCTC events.	

Theme: Recommendations for questionnaire and for those completing it

Category: Questionnaire developments	Informant
I would only maybe include some questions about sponsorship in internet or sponsorship in traditional media like tv, radio, newspapers… in that… because in {named country} we have that in social media... regulated. Not as strong as we might want to.	400
If you want to help focal points in countries to answer the survey fluently… rapidly, you have to translate it in French. It is possible.<br><br>Many guidelines are in French. If we have guidelines in English, I think it is better to translate them… and share them with the countries.<br><br>The interface of the survey, for me it is important to give it to the Secretariat to have a translation on the website. It is one of the reasons that I accepted to participate in this interview. Because, do not forget that I am {named identifiable held position}. And I have an ear of my colleagues in other countries…francophone. They have the same difficulty for reporting. Reporting is very easy, but you have to understand the questions. If you have problem to understand the question, try maybe to translate the question. For that reason, I know the staff of the Secretariat… to give my experience… I said yes [to participate in the interview].	614
If they could provide with the answer for the… especially in the policy… if it is clearly stated that cross-border promotion or at point of sale… is totally banned… so we can easily answer to this. INT: What would it be better then? To have a more clear question, or a more clear regulation?<br>PA: A more clear regulation.	702
Maybe to actually… to write the question easier… Maybe we have to change the question, because it is hard when we need to approve the government authorities and to report any response to this question regarding the government [decision]… it is not… we need to look for other questions that get the result for this question.<br><br>INT: Please, remind me again to which question are you referring to?<br>PA: Actually, my observation on this question is that we need to develop a question that can actually answer this current situation for the government authority…	1129

Any expenditure of the tobacco industry… it needs more clarification for this question.<br><br>My proposal is to divide it in two parts. One part is about the legal reference of prohibition of expenditures of the tobacco industry and the other part [should be] if it is done so… [is there] any example of such kind of tobacco industry expenditures on advertising, promotion and sponsorships. For example… putting cigarettes at point of sale are example of tobacco industry's expenditures on tobacco advertising. Usually the tobacco industry align… do expenditures at the point of sale. Ban in practice we ban it. So any respond to this question depends on the… actually… how can I say… understanding this question… so we need to have more clarification on this question… to have a uniform response.	
What do you believe that could help in order to make it a little bit more… clear?<br>PA: … Maybe it can be "what type of tobacco advertisement, promotion and sponsorship entering your territory for which domestic regulation apply"… maybe… "what type"? … to ask what type… or "the same form"… "Is the same form"?...<br><br>Yes, yes… yeah. It would make it more clear, because it would also prevent about that… going back to the top… to link it to the [question]… yeah. The right way with it is… that it makes it very easy… very simple. But if you put it there too it makes it straight forward. But where it is… Now I understand it very well. I think you can either leave it as it is or put it there… If you put it there it makes the flow easy. If you do not put it there too… because whoever is reading it and makes the link to the top you can easily get the answer to.<br>INT: That's great.<br>PA: For me, if you say that "ban covers"… yes, it will flow... you put it there. For this particular question, if you put this "your ban covers" it will make it more easy… well understandable for the person who reads it and the answer can be straight forward.<br><br>It is only that particular clause that I do not understand it very well. If that particular clause was added close… "does your ban covers"… you continue the same… But the way it is, to me it is very fine, is very clear and is very understandable. Because when I was reading this it was very fine… because if it was straight forward I would understand it very well. That is me. That particular one… that's why I put that… when I was asked I was on it… not the one I submitted to you…Because I was not fully understood… But the way you show to me… you linked those two with the ban cover… it is very fine, it flows very well. To me, I think it is ok with me as it is.	1239
I would suggest that definition and additional instructions for specific questions. It will be help for answers exactly as the questioner require.<br><br>From the answer in Question No8. that I would suggest for definitions and additional instructions for clearly querstion as follows,<br>1. contributions from tobacco companies to any other entity for "socially responsible causes" and/or any other activities implemented under the umbrella of "corporate social responsibility" by the tobacco industry?<br>2. cross-border advertising, promotion and sponsorship originating from your territory?<br>3. the same forms of cross-border advertising, promotion and sponsorship entering your territory for which domestic regulation apply?	1335
There is a lot of information. And I think it should be some more responding options rather than just a simple "yes" or "no".<br><br>When you look this size… this is one aspect of the FCTC report. I think that's quite a lot of questions already. So, I do not think anything more needs to be added in.	1827
My question is how the question C273 links to the other [following] questions? So, I propose in as in question C271… there is a line between question C271 and question	1839

C272. That explicitly explains that if you answered “no” or “no” [here is] how to proceed. Probably, such explanations between C273 [and the following ones] should also be presented.<br><br>So probably this explanation should also be reformulated…. between C271 and C272… it should be reformulated.<br><br>INT: What is your suggestion? How should it look like?<br>PA: So, the question C271 is OK, it could stay as it is. To my understanding, regarding C271 and C272 is better to remove all restrictions… [the restriction that says] “if you answered “yes”…” or if “no” then skip [section C272]. My opinion is that all the questions [under section C272] should be answered and not depending on how the question C271 is answered.<br><br>“Are you precluded by your constitution or constitutional principles from applying restrictions on all tobacco”! This is the correct! So my proposal would be probably to remove the question C273 totally from the questionnaire… and at all the following questions the statement about the constitutional precludes. Because question C273, somehow becomes redone. Or probably to not rephrase all the questions… [but] at the beginning of the block you have to add some kind of explanation to reach [an understanding] about the content of the questions…. [to showcase that] they are related to the constitution… or how these questions should be considered…. how they should be answered… in which context.<br><br>INT: So you are saying that there are some difficulties of understanding the word “cooperation”? What does it mean? Does it meet discussions? Does it mean…<br>PA: OK if we change the “cooperating”… to your understanding, to what should be changed?<br>INT: Yeah… Actually, that is my question!<br>PA: “Activities” maybe… “activities” or… “cooperating” is more… [for] higher level. “Activities”… maybe “activities” or “discussions” or “dialogues”… we need a word for this… Maybe “activities”… “Any activities… with other Parties”. Yes… yes!<br><br>So, probably we need to pay more attention on how the questions are formulated, so they can be more… transparent to us. One of the approaches is… before sending the questions to us to fill them in, you maybe insight the organization and do [so some] kind of piloting of the questionnaire. Try to share it with somebody who is in the field and can provide you feedback. [Like] the way we are doing it right now… during the current interview. We are usually doing this [approach] in our department… [when] creating every survey or whatever we pilot the questionnaire. We get the feedback back and then we make any necessary changes to the questionnaire.	
**Category: Personal and organisational development**	
Well… I have not thought about that before… but it would be like a good recommendation that we will have to follow… to create a document to… do easy the job for the people who want to know about all the information… legal or not legal. That would be good. I haven’t thought about it. Maybe no one has thought about that in the office… I will tell them!	400
Ah OK. I think they have to see the question about… implementation… the Guidelines. Because if they do not know about the Protocol and the Guidelines, it is not easy to… maybe they will have some difficulties to answer to this question… in my opinion.<br><br>I think my suggestion is to have a meeting for francophone countries’ national focal points and to explain how to use the website and how to use easily this interface in this language. You need to have good data [entry]. Many countries have problem to answer	614

the reporting in the web, you know. May countries they do not know how to use the website… the data. If maybe… if you organise one meeting… a workshop with them… that would help to have good data on time.<br><br>That's why I think sometimes it is important to… when we say to make the meeting to share the information… for capacity building.	
I think I need to learn more and to gain more experience because three and a half years… because we do not have any specific training because of the pandemic COVID-19… that's how things been led here so… I think more sharing sessions… more training will work for me.<br>INT: A training within the team, or do you prefer some additional information from the FCTC side?<br>PA: For my team, but also from the FCTC.<br><br>They should be trained more in the… I mean at the regulations as well, because are having so many regulations from the custom… from the trade, I mean from industry… from other sectors as well, so they should be trained as a whole, so they can answer clearly and… maybe for the policy… it would be better for the policy development actually.<br><br>Maybe for the… because cross-border is also involved… the Ministry of Trade and also the Custom… if there is anything related to the industry or any promotion of the products they should be considered as well. We should collaborate with them to prevent from being advertising… all tobacco products… in our country. So we should to… check in which areas promotion can be done or cannot be done.<br><br>I think more discussion between the agencies should be conducted to answer things we do not know… I think we need to conduct more meetings and also discussions actually.<br><br>But for certain things we should strictly stick in our regulation… we definitely… it is easy for me to understand.	702
I think they do not think about it…. Cooperating with other countries to eliminate cross-border advertisement. They do not think… or they do not teach… Maybe it is because of the national focal points. They have to raise the issue with other countries… so it is the responsibility of the focal points to be very sensitive for this issue and to aired the relevant agencies… to the national authorities to be sensitive for this issue, and then, for example an expert group to be established in the judicial system to… to Attorney General… an authority… They have to develop any guidance, any operational guidance for that work… and raise any issue for the legal system to be legalised… to legalise the action. So it is the legal institution to… institutionally actually develop any legal infrastructure… it is very important at the country level. Then, [after the] establishment the legal infrastructure we can use this power to intervene. And also we need to have cooperation with other countries to the [level of] Ministry of Foreign Affairs, because it is very important to have some… policies on this issue. This is my answer to you.<br><br>Yes, FCTC can raise the issue, but mainly the Parties should raise the issue at the COP level. It is very important to have a solution at COP for any issues that... for example on this issue they have to submit a draft decision to the COP, to solve the problem of cooperation of the countries… the Parties. It is a very important topic.<br><br>So, any question that the Parties answer in this questionnaire should refer to the legal system… if any country have no national legal reference to respond to the question, they may answer differently from other countries…maybe… maybe. But if we have the Guidelines for the national legal system… it is easy to answer. But in case... for example "requiring the disclosure to relevant Government authorities of expenditures by the	1129

tobacco industry on advertising,"… it is prohibited, but not directly about tobacco.<br><br>I do not know, but my proposal is to involve WHO office in every country… to be involved in the process of the report. For example they maybe… can just select the new focal point for the system… so maybe he [can] receive any information… so we need to have fundamental cooperation with the WHO office at country level.<br><br>Eh… to facilitate the reporting practice, I think the main issue is to have actually the infrastructure… and have coordination mechanisms at national level… to coordinate with all the relevant agencies. It is very important to have a such a kind of cooperation system with other agencies. In case of not having any coordination mechanism it is not easy for the national focal point to do this task… very easy. So, in any case the countries which do not have a coordination mechanism they have a problem… and all the responsibility is on the national focal point to do [the reporting] individually. But my proposal for these countries is to use the power of the WHO [country] office… to intervene and help the national focal point… to actually help… assist the national focal point to develop and complete the questions. The process of completion is very important… and my proposal… my final proposal is that WHO… I know the governing body of the WHO and the Secretariat are not working together... actually the governing body is different from the WHO [FCTC] Secretariat, so it is not easy, but maybe a memorandum of understanding… such kind of mechanism is required to verify the reporting instrument developed by the national focal points. We need to have such a kind of system because in {named country} we have [such a system] and we proposed other Parties to have this kind of verification mechanism.<br><br>So, I think the national focal point… I advise the national focal point as soon as they receive the… because usually the questions are the same… each year. So every year the questionnaire is unique and has the same questions we ask for the other agencies… so we need to send a formal letter because annually they [have to] complete the [same] questions… so it just needs for every focal point to send the questionnaire to the agencies six months before the reporting period comes. So it is easy… it facilitates the work of the national focal points. It depends on the national focal point how to do this… how to follow up with the issue and facilitate or work with completing the reporting instrument. So it depends on the national focal point to do any intervention to facilitate with his or her work on this important issue.	
I would like that... a training on it, rather than just two days – three days – one week training… is never enough.<br><br>Training… training is something ongoing… and it depends on the individual. Because my understanding and your understanding and someone's else understanding on these questions might be different. I am not new in the tobacco control. So most of these questions would be very easy for me, but for like somebody coming new to the tobacco control, maybe it would be very difficult for the person to answer these [questions] within a short possible time or maybe will not be aware of the things [mentioned] here. So it needs training. So, I think it depends on the particular individual you are dealing with. But, trainings for this… it is not necessary for me… Generally on tobacco control is fine. Because I have never had a formal training on tobacco control. But on this in particular… I think I am ok with this. Because as I said earlier, it depends on the individual.<br><br>Yeah, in general they need to be trained on different articles of the tobacco control. Not only on this Article 13, but all the different articles. Because we are dealing with article 13, let me limit myself on a different term. I think there is a need for a taught training so that people can be offered with… and also provide the materials given sometimes… you just take them but you do not have the time to read these, but if there can be small	1239

trainings there and there… the person can refresh his memory… his mind on some of those things. Or just [by] printing the documents or sending them by email… you have other things to do… you just sometimes keep them in your emails and never download them. If there is at least a refresh training, you call people to [join] or you can even do it online. You can refresh these memories on these Articles. It is very important… very important. But it is rather… sometimes difficult… or [people do] not [join] as expected…. There are some tobacco control trainings but there are not as many [people] as expected.	
I will write the step by step guidance on completing the FCTC report for my successor.	1335
Eh… I think, as I was new at that time, it would really help if… I mean in that situation… if I had a more organised and structured unit for… you know for the tobacco control area, where we would have regular reporting from other agencies, so they would be feeding us with information on a regular basis…. And how to set the data. That would be really-really good, but also to explore further resources that… I think it is very difficult right now to be able to…. to set up. We are gradually getting there… to the solution in terms of health systems strengthening model where we are continuing the various focal points and expertise in place, but the unit is just one person… in these new areas. So, you know it is very difficult. I think as I become more experienced with it… I [have done] two cycles… two reports already… so I have kind of set up my own networking and my own understanding… my own awareness of where I need to go… to get the data sources… the best agencies to work with them… the persons who I have established a network who are willing to assist. But I think what I would like to see… where I would like to see going with this is a structured tobacco control unit or NCDs unit with the proper resources and the personnel to better coordinate.<br><br>It is a lot of work, but as I said before, I think it would be really helpful if we have a unit set up for tobacco control with staff... individuals with specific responsibilities of the key areas of tobacco control.	1827

Consolidated criteria for reporting qualitative studies (COREQ): 32-item checklist

Adapted from:

Tong A, Sainsbury P, Craig J. Consolidated criteria for reporting qualitative research (COREQ): a 32-item checklist for interviews and focus groups. *International Journal for Quality in Health Care*. 2007. Volume 19, Number 6: pp. 349 –357

	**Comment**	**Location in manuscript**
**Domain 1: Research team and reflexivity**		
Personal Characteristics		
1. Inter viewer/facilitator: Which author/s conducted the interview or focus group?	Lead author	Information removed from final manuscript due to word count limitation
2. Credentials: What were the researcher's credentials? E.g. PhD, MD	Doctorate student	Information removed from final manuscript due to word count limitation
3. Occupation: What was their occupation at the time of the study?	Doctorate student	Information removed from final manuscript due to word count limitation
4. Gender: Was the researcher male or female?	Male	Information removed from final manuscript due to word count limitation
5. Experience and training: What experience or training did the researcher have?	Trained at master's studies level	Information removed from final manuscript due to word count limitation
Relationship with participants		
6. Relationship established: Was a relationship established prior to study commencement?	No	N/A
7. Participant knowledge of the interviewer: What did the participants know about the researcher? e.g. personal goals, reasons for doing the research	An introduction was provided by the FCTC Secretariat alongside the study's purpose and invitation for participation. There, the Participants also learned about the members of the PhD supervisory team and that lead author would be undertaking the interviews for the study.	Information removed from final manuscript due to word count limitation

<u>8. Interviewer characteristics:</u> What characteristics were reported about the interviewer/facilitator? e.g. Bias, assumptions, reasons and interests in the research topic	Under the study's purpose was reported that the aim was to improve the FCTC questionnaire. Also, a researcher's self-introduction was given at the beginning of the interview.	Information removed from final manuscript due to word count limitation
**Domain 2: study design**		
Theoretical framework		
<u>9. Methodological orientation and Theory:</u> What methodological orientation was stated to underpin the study? e.g. grounded theory, discourse analysis, ethnography, phenomenology, content analysis	Thematic framework analysis without underpinning theory	Page 6, line 5
Participant selection		
10. Sampling: How were participants selected? e.g. purposive, convenience, consecutive, snowball	Purposive sampling & selection criteria	Page 4, line 31-32 & Page 45 lines 2-6
<u>11. Method of approach:</u> How were participants approached? e.g. face-to-face, telephone, mail, email	Email	Page 5, line 1
<u>12. Sample size:</u> How many participants were in the study?	Nine	Page 5, lines 20
<u>13. Non-participation:</u> How many people refused to participate or dropped out? Reasons?	Twenty-five	Page 5, lines 8-18
Setting		
<u>14. Setting of data collection:</u> Where was the data collected? e.g. home, clinic, workplace	Online via Microsoft Teams	Page 5, line 33
<u>15. Presence of non-Participants:</u> Was anyone else present besides the participants and researchers?	No	N/A
<u>16. Description of sample:</u> What are the important characteristics of the sample? e.g. demographic data, date	Section about participants' characteristics	Page 5, lines 20-24
Data collection		

17. Interview guide:<br>Were questions, prompts, guides provided by the authors? Was it pilot tested?	Interview guide provided in supplementary fine – pilot tested	Page 5, lines 31-32
18. Repeat interviews:<br>Were repeat inter views carried out? If yes, how many?	No repeat interviews because too much burden for participants	N/A
19. Audio/visual recording:<br>Did the research use audio or visual recording to collect the data?	Audio recording via digital dictaphone	Page 5, line 33
20. Field notes:<br>Were field notes made during and/or after the interview or focus group?	No	N/A
21. Duration:<br>What was the duration of the inter views or focus group?	Average duration 49min	Page 5, lines 24-26
22. Data saturation:<br>Was data saturation discussed?	Discussed during study design, however no more participants could be reached as they have ignored or rejected our invitation	N/A
23. Transcripts returned:<br>Were transcripts returned to participants for comment and/or correction?	Participants were questioned for receiving the manuscripts. Only one requested them to validate the content	Information removed from final manuscript due to word count limitation
**Domain 3: analysis and findings**		
Data analysis		
24. Number of data coders:<br>How many data coders coded the data?	Two first authors	Page 6, lines 5-6
25. Description of the coding Tree:<br>Did authors provide a description of the coding tree?	Tables 2 and 3	Page 6, lines 9-26
26. Derivation of themes:<br>Were themes identified in advance or derived from the data?	From the data	Page 6, lines 6-26
27. Software:<br>What software, if applicable, was used to manage the data?	NVivo, version 12	Page 6, line 14
28. Participant checking:<br>Did participants provide feedback on the findings?	FCTC Secretariat and Participants were informed about the study findings	Information removed from final manuscript due to word count limitation
Reporting		

29. Quotations presented:<br>Were participant quotations presented to illustrate the themes/findings? Was each quotation identified? e.g. participant number	Yes	Reported in manuscript (Results section Pages 7-11) and in Supplementary File 3
30. Data and findings consistent:<br>Was there consistency between the data presented and the findings?	Consistency was evaluated by the first two authors as part of the analysis and then by all authors during manuscript drafting	Page 6, lines 24-28
31. Clarity of major themes:<br>Were major themes clearly presented in the findings?	Clarity presentation was evaluated by all authors	N/A
32. Clarity of minor themes:<br>Is there a description of diverse cases or discussion of minor themes?	N/A	N/A

© 2023 Tselengidis A. et al.
